# Targeted Suppression and Knockout of ASCT2 or LAT1 in Epithelial and Mesenchymal Human Liver Cancer Cells Fail to Inhibit Growth

**DOI:** 10.3390/ijms19072093

**Published:** 2018-07-19

**Authors:** Paige J. Bothwell, Clare D. Kron, Evan F. Wittke, Bradley N. Czerniak, Barrie P. Bode

**Affiliations:** Department of Biological Sciences/Center for Biochemical and Biophysical Studies, Northern Illinois University, DeKalb, IL 60115, USA; pbothwell1@niu.edu (P.J.B.); clarekron@frontier.com (C.D.K.); ewittke@luc.edu (E.F.W.); bczerniak2@hotmail.com (B.N.C.)

**Keywords:** ASCT2, SLC1A5, LAT1, SLC7A5, hepatocellular carcinoma, CRISPR-Cas9, shRNA, amino acid transport, mTOR

## Abstract

Amino acid transporters alanine-serine-cysteine transporter 2 (ASCT2) and L-Type Amino Acid Transporter 1 (LAT1) are coordinately enhanced in human cancers where among other roles, they are thought to drive mechanistic target-of-rapamycin (mTOR) growth signaling. To assess ASCT2 and LAT1 as therapeutic targets, nine unique short hairpin RNA (shRNA) vectors were used to stably suppress transporter expression in human epithelial (Hep3B) and mesenchymal (SK-Hep1) hepatocellular carcinoma (HCC) cell lines. In addition, six unique CRISPR-Cas9 vectors were used to edit the ASCT2 (*SLC1A5*) and LAT1 (*SLC7A5*) genes in epithelial (HUH7) and mesenchymal (SK-Hep1) HCC cells. Both approaches successfully diminished glutamine (ASCT2) and leucine (LAT1) initial-rate transport proportional to transporter protein suppression. In spite of profoundly reduced glutamine or leucine transport (up to 90%), transporter suppression or knockout failed to substantially affect cellular proliferation or basal and amino acid-stimulated mTORC1 growth signaling in either HCC cell type. Only LAT1 knockout in HUH7 slightly reduced growth rate. However, intracellular accumulation of radiolabeled glutamine and leucine over longer time periods largely recovered to control levels in ASCT2 and LAT1 knockout cells, respectively, which partially explains the lack of an impaired growth phenotype. These data collectively establish that in an in vitro context, human epithelial and mesenchymal HCC cell lines adapt to ASCT2 or LAT1 knockout. These results comport with an emerging model of amino acid exchangers like ASCT2 and LAT1 as “harmonizers”, not drivers, of amino acid accumulation and signaling, despite their long-established dominant role in initial-rate amino acid transport.

## 1. Introduction

The cancerous transformation of cells and the subsequent stages of tumorigenesis involve a metabolic and physiological reprogramming to support constitutive growth. Such derangements include enhanced glucose uptake and aerobic glycolytic metabolism (“the Warburg effect”), as well as accelerated glutamine transport and glutaminolysis, characterized as “glutamine addiction” [1,2,3,4]. While these physiological aspects of cancer have been recognized for decades, there is currently a renewed interest in their molecular underpinnings in the hope of finding novel therapeutic strategies.

Cellular metabolism within a tumor microenvironment is subject to fluctuating nutrient conditions as well as active communication and metabolite exchange between tumor and stromal cells [5]. Likewise, nutrient transporters play an important role in fueling the dynamic metabolic economies within tumors. The advancement of genome and transcriptome sequencing tools have led to the creation of vastly informative databases such as the Cancer Genome Anatomy Project (CGAP), the Cancer Genome Atlas (TCGA), and the Cancer Gene Expression Database (CGED), among others, and these data assemblies have been critical to the investigation of potential therapeutic targets in tumor metabolism [6,7,8,9]. In 2005, the human expressed sequence tag (EST) database from the CGAP was used to identify a coordinately enhanced expression of two amino acid transporters, alanine-serine-cysteine transporter 2 (ASCT2) and large neutral amino acid transporter 1 (LAT1), across a variety of human cancers relative to normal tissue [10]. At the time, this finding was consistent with enhanced functions of both transporters in cancerous vs. normal cells [11,12].

The ASCT2 and LAT1 amino acid transporters function in a cooperative, tertiary antiporter mechanism (Supplemental Figure S1). ASCT2 is a broad specificity Na^+^-dependent antiporter (exchanger) which exploits the electrochemical gradient of sodium across the plasma membrane to exchange one small, neutral amino acid from the cytoplasmic side for one small, neutral amino acid and a sodium ion on the extracellular side. LAT1 also functions as an antiporter, but its mechanism is Na^+^-independent. In the tertiary active transport model, LAT1 utilizes the intracellular pool of amino acids in part generated by ASCT2 activity to exchange one small, neutral amino acid from the cytoplasmic side for one large, essential amino acid from the extracellular side. This cooperative mechanism was suggested to provide a rationale for the coordinate upregulation of both transporters in cancer [10]. The model was reinforced by the concurrent discovery that CD147 and CD98hc nucleated a “metabolic activation-related complex” with LAT1, ASCT2, and monocarboxylate transporters (MCT) in the plasma membrane of cancer cells [13]. As ASCT2 and LAT1 ostensibly equilibrate intracellular amino acid pools through exchange mechanisms that are responsive to metabolic demands, such transporters have recently been designated as “amino acid harmonizers” by Bröer et al. [14].

Why the ASCT2 and LAT1 partnership? In the tertiary active transport/harmonizer model, the complementary transport activities of ASCT2 and LAT1 sustain an intracellular pool of essential amino acids that ostensibly stimulates the activity of mammalian (now, mechanistic) target-of-rapamycin (mTOR) growth signaling complex 1 (mTORC1), a serine threonine kinase which is a critical activator of cap-dependent protein translation and also acts to inhibit autophagy (Supplemental Figure S2). More than a decade ago, studies from our lab [15] and others [16] initially implicated ASCT2 in mTORC1 signaling, which helped prompt more recent investigations into the role of these two transporters in cancer. Subsequent studies showed that ASCT2 and LAT1 also function in, myc-driven transcriptional programs, mitochondrial glutamine metabolism, and glycolytic diversion [15,16,17,18,19]. Thus, their potential as therapeutic targets has been well founded to-date.

ASCT2 has been targeted with shRNA and small molecular inhibitors in a broad spectrum of cancers including melanoma [20], prostate cancer [21], human head and neck squamous cell carcinoma [22], and breast cancer [23], among others. These studies generally found that suppression of ASCT2 led to detrimental effects on glutamine transport, mTORC1 signaling, proliferation, and cell cycle progression. LAT1 has also been targeted with shRNA and small molecular inhibitors in a broad spectrum of cancers including esophageal squamous cell carcinoma [24], gastric cancer [25], cholangiocarcinoma [26], breast cancer [27], ovarian cancer [28], and endometrial carcinoma [29], among others. Like ASCT2, these studies generally found that the suppression of LAT1 led to detrimental effects on leucine transport, mTORC1 signaling, proliferation, and cell cycle progression. More recent studies with ASCT2 or LAT1 knockout cancer cell lines suggest a more complex role for these transporters in tumorigenesis [14,30].

Previous work from our lab with an inducible long antisense RNA system clearly implicated ASCT2 as a viable therapeutic target in human HCC cells [31]. Here, those studies were extended using viral shRNA encoding vectors and the CRISPR-Cas9 gene editing system because they offer more specific and tractable modalities for in vivo applications. Both approaches were pursued and reported here because recent studies have yielded contradictory results with genes implicated in cancer targeted by RNA interference or CRISPR-Cas9 mediated gene editing [32]. Given the enhanced expression of ASCT2 and LAT1 in HCC, the studies presented here were designed to assess the therapeutic potential of targeting each transporter, and to determine whether primary (epithelial) or metastatic (mesenchymal) phenotype human HCC cells are differentially reliant on these transporters for growth and viability.

Contrary to expectations, the results from both approaches contradicted earlier studies with inducible long-antisense RNA [31]. While shRNA and particularly CRISPR-Cas9 profoundly suppressed glutamine and leucine initial-rate transport, the results indicated that neither suppression nor knockout of ASCT2 or LAT1 is alone sufficient to inhibit the in vitro growth of epithelial or mesenchymal human HCC cells, or to suppress mTORC1 signaling. Potential reasons for the conflicting results with long antisense RNA and suggestions for a path forward in this research area are discussed in light of our findings and recent results from ASCT2 and LAT1 knockout studies in other types of cancer cells.

## 2. Results

Note: in the main text and supplemental figures, data from the epithelial cell lines (Hep3B and HUH7) are shown in red, and data from the mesenchymal cell line (SKHep) are shown in blue. All data for the shRNA-based work from the first half of this study can be found in the Supplemental Materials, and a description of these findings is provided first, in Results Section 2.1 and Section 2.2.

### 2.1. shRNA-Mediated ASCT2 and LAT1 Suppression Reduces Amino Acid Transport, but Fails to Affect Proliferation Rate

Hep3B and SKHep cell lines were transfected with unique short hairpin RNA/micro RNA (shRNAmir) plasmid vectors, and then grown under puromycin-selective conditions. No clonal isolation was performed after antibiotic selection. Stable cell populations that were positive for the vector’s visual reporter, green fluorescence protein (GFP), were imaged using both transmitted light and a green fluorescence excitation channels, and these images were digitally superimposed (Supplemental Figures S7 and S8). The knockdown of ASCT2 and LAT1 amino acid transporter mRNA was assessed by reverse transcriptase-quantitative polymerase chain reaction (RT-qPCR) analysis (Supplemental Figure S9). In general, suppression of ASCT2 mRNA expression was more robust in stably transfected SKHep, relative to Hep3B. Overall, shRNA-based targeting of ASCT2 transporter mRNA reduced its expression by 15–33% in Hep3B and 49–96% in SKHep (Supplemental Figure S9, A vs. C), and shRNA-based targeting of LAT1 mRNA reduced its expression by 63–85% in Hep3B and 66–93% in SKHep (Supplemental Figure S9, B vs. D).

Western blot analysis was used to measure changes in ASCT2 and LAT1 transporter protein expression across the stably transfected Hep3B and SKHep cell lines (Supplemental Figures S10 and S11, respectively). The most effective shRNAmir designs were as follows: in Hep3B, the A2 and A5 shRNAmir sequences reduced ASCT2 protein expression by 40% and 25%, respectively, and the L1 and L3 shRNAmir sequences reduced LAT1 protein expression by 84% and 86%, respectively (Supplemental Figure S10). In SKHep, the A2 and A5 shRNAmir sequences reduced ASCT2 protein expression by 85% and 44%, respectively, and the efficacy of all four LAT1 shRNAmir sequences was comparable, ranging from 80% to 92%. However, L1 and L3 were again the most effective, generating 90% and 92% knockdown of LAT1 protein, respectively (Supplemental Figure S11).

Initial rate amino acid uptake analysis was performed using ^3^H-radiolabeled l-glutamine and l-leucine to assess the functional efficacy of transporter suppression. Overall, the pattern of reduction in Na^+^-dependent initial rate glutamine uptake activity across the ASCT2-targeted Hep3B and SKHep cell lines (HA1–HA5 vs. SA1–SA5, respectively) correlated to the observed patterns of ASCT2 protein knockdown from Western blot analysis (Supplemental Figure S12A,B). These results indicate that Hep3B and SKHep cells did not compensate to restore their former rates of glutamine uptake. In LAT1-targeted Hep3B cell lines (HL1–HL4), initial rate leucine uptake rates were refractory to, or differentially affected by LAT1 suppression, although the leucine uptake activity in the HL1 and HL4 cell lines was significantly reduced (Supplemental Figure S12C). Compared to Hep3B, the LAT1-targeted subset of SKHep cell lines (SL1–SL4) demonstrated more substantial reductions in leucine uptake rate (Supplemental Figure S12D), consistent with the reduced levels of LAT1 protein observed in Western blot analysis (Supplemental Figure S11).

According to the current model for the ASCT2 and LAT1 transport mechanism, these two transporters function cooperatively as obligatory amino acid exchangers whereby substrates (e.g., glutamine) provided by ASCT2 fuel the exchange mechanism of LAT1 for the import of essential amino acids—termed “tertiary active transport” [10,16]. Therefore, initial rate transport analysis was performed to determine if ASCT2 or LAT1 suppression exhibit mutual effects on each other’s activity (Supplemental Figure S13). The results differed depending on the parent cell line, Hep3B or SKHep, and though the results were statistically significant, all of these changes were relatively minor in scale (≤20%).

Subsequent RT-qPCR analysis did not show a compensatory upregulation of alanine-serine-cysteine transporter 1 (ASCT1) mRNA expression in either ASCT2- or LAT1-targeted hepatoma cell lines (Supplemental Figure S14A,B). This result contradicts an early study which used an inducible antisense ASCT2 targeting system in SKHep, and that work demonstrated that ASCT2 knockdown induces an increase in ASCT1 expression [31]. Furthermore, RT-qPCR analysis also revealed that sodium-dependent neutral amino acid transporter 2 (SNAT2) and glutamine synthetase (GLUL) mRNA expression were also not substantially changed after ASCT2 and LAT1 were suppressed via shRNAmir (Supplemental Figure S14C–F). The expression of large neutral amino acid transporter 2 (LAT2) mRNA was also analyzed via RT-qPCR, but LAT2 mRNA was undetectable in both Hep3B and SKHep parent cell lines as well as in their derivative transfected cell lines. Interestingly, two SKHep-derived cell lines, ASCT2-targeted SA2 and LAT1-targeted SL1, consistently demonstrated a four-fold increase in cystine-glutamate transporter (xCT) mRNA expression (Supplemental Figure S14H). However, Western blot analysis revealed no change in xCT protein expression in the same cell lines; and this observation was reinforced later when ASCT2 and LAT1 were silenced again with CRISPR-Cas9 in HUH7 and SKHep hepatoma cell lines (Supplemental Figure S25).

Cellular proliferation rate was assessed by methylthiazol tetrazolium (MTT) analysis (Supplemental Figure S15). Proliferation rates in both the Hep3B and SKHep ASCT2 and LAT1 suppressed cell lines were statistically variable, but not substantially altered from the parent cell line and nonsilencing controls. RNA interference (RNAi)-based suppression of ASCT2 and LAT1 transporter mRNA and protein expression, and cognate reductions in amino acid initial rate uptake, together were not sufficient to significantly affect cellular proliferation.

### 2.2. shRNA-Mediated Repression of ASCT2 and LAT1 Fails to Affect mTORC1 Signaling

Cellular growth is influenced by the integration of extracellular nutrient and growth factor signals, and an essential hub of this signaling cascade is mTORC1, a serine threonine kinase activated by amino acids [33]. When active, mTORC1 is phosphorylated and functions to activate cap-dependent protein translation and inhibit autophagy by phosphorylating numerous downstream targets, including 70 kDa ribosomal protein S6 kinase (p70 S6K), eukaryotic initiation factor 4E (eIF-4E) binding protein 1 (4E-BP1), and unc-51 like autophagy activating kinase 1/2 (ULK1/2) (Supplemental Figure S2). To assess the impact of shRNA-mediated ASCT2 or LAT1 suppression in mTORC1-mediated growth signaling in the Hep3B and SKHep cell lines, Western blot analysis was performed to evaluate the phosphorylation states of two mTORC1 targets: 4E-BP1 and p70S6K—under controlled conditions of growth factor stimulation, amino acid availability, small molecular amino acid transport inhibition, and rapamycin treatment as indicated in the schema depicted in Supplemental Figure S16. No differences in the phosphorylation levels of 4EBP1 or p70S6K were observed between transporter suppressed lines and their cognate controls under normal growth conditions—although SKHep exhibited more robust 4EBP1 phosphorylation than Hep3B, reflecting its more aggressive growth phenotype (Supplemental Figure S17A; p-4E-BP1^T37/46^ and p-p70S6K^T389^).

Likewise, both Hep3B and SKHep and their transporter suppressed derivatives displayed equal sensitivity to the removal of serum (termination of growth factor-dependent signaling), although the Hep3B lines were more sensitive than SKHep, manifest by more profound reductions in 4EBP1 upper band phosphorylation (Supplemental Figure S17B; p-4E-BP1^T37/46^ and p-p70S6K^T389^). Basal mTOR1 signaling (under conditions of amino acid- and serum-deprivation) likewise exhibited no differences between the parent lines and their transporter-suppressed derivatives (Supplemental Figure S18A; p-4E-BP1^T37/46^ and p-p70S6K^T389^). Repletion of amino acids (both glutamine and essential amino acids) in the absence or presence of ASCT2 or LAT1 inhibitors also failed to display any correlation of mTORC1 signaling readouts to transporter suppression (Supplemental Figures S18 and S19) or differences in sensitivity to rapamycin (Figure S20). In summary, surprisingly, the Hep3B and SKHep cell lines stably suppressed for ASCT2- and LAT1 expression did not demonstrate aberrant mTORC1 signaling or unique nutrient sensitivities compared to nonsilencing and parent control lines. The data suggest that the cells adapted to the suppressed amino acid delivery—an outcome that aligns with the sustained growth rate phenotype demonstrated by these ASCT2 or LAT1 deficient cell lines.

### 2.3. CRISPR-Cas9 Effectively Knocks out ASCT2 and LAT1 Expression, But Does Not Elicit Cell Death in Epithelial (HUH7) or Mesenchymal (SKHep) Liver Cancer Cells

The strategy of using shRNA to suppress ASCT2 and LAT1 transporter expression yielded only a partial repression of the two transporters. Therefore, the CRISPR-Cas9 gene editing system was employed to introduce permanent, dysfunctional mutations to the ASCT2 and LAT1 genes (SLC1A5 and SLC7A5, respectively). The hepatoma cell line HUH7 was used instead of the Hep3B cell line, as representative of epithelial-type HCC due to the difficulty of successfully transfecting Hep3B with the CRISPR-Cas9 plasmid vectors. HUH7 and SKHep cells were each transfected with CRISPR-Cas9 vectors encoding three unique guide RNA (gRNA) sequences against the SLC1A5 gene (A1, A2, and A3), three unique gRNA sequences against the SLC7A5 gene (L1, L2, and L3), and one gRNA sequence that served as a nonsilencing system control (NS). All derivative cell lines were grown under puromycin-selective conditions. No clonal isolation was performed after antibiotic selection, and therefore, the resultant cell populations contain a mixture mutational variants resulting from the CRISPR-Cas9 activity.

**Figure 1:**
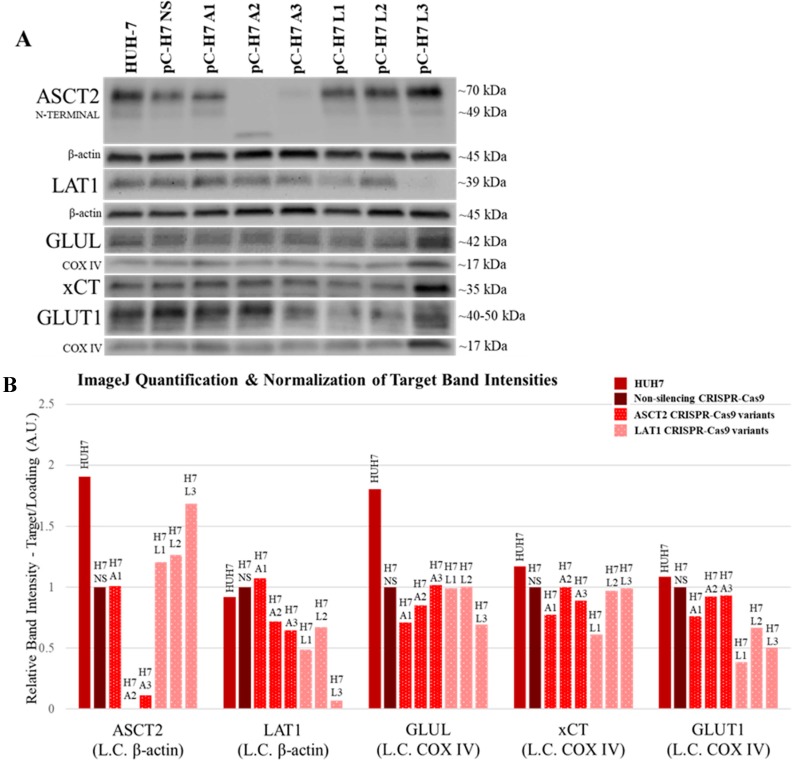
ASCT2, LAT1, GLUL, xCT, and GLUT1 protein expression in HUH7 cell lines targeted for gene knockout with CRISPR-Cas9. Protein expression was measured by Western blot analysis as described in Materials and Methods. (**A**) The chemiluminescent bands for each target (ASCT2, LAT1, GLUL, xCT, and GLUT1) are shown in comparison to β-actin or COXIV protein detected on the same blots to control for loading error. One image for COXIV is shown for both xCT and GLUT1 because these proteins were detected on the same blot; (**B**) the band intensity of each target was quantified using ImageJ analysis software and normalized to loading control (L.C.) band intensity in each lane.

Western blot analysis revealed that in HUH7, the ASCT2-targeted pC-H7 A3 and the LAT1-targeted pC-H7 L3 cell lines demonstrated the most successful knockouts of the ASCT2 and LAT1 proteins, respectively (Figure 1). Remarkably, transfection with the A2 CRISPR-Cas9 plasmid construct led to a robust knockout of ASCT2 accompanied by the appearance of a currently unidentified, lower molecular weight band that was reactive with ASCT2 antibody (Figure 1A). Glutamine synthetase (GLUL) and cystine-glutamate transporter (xCT) protein expression were both unaffected by ASCT2 and LAT1 transporter knockout (Figure 1, GLUL and xCT). Interestingly, glucose transporter 1 (GLUT1) protein expression was consistently reduced by ~50% in the three LAT1 targeted cell lines, pC-H7 L1–L3 (Figure 1, GLUT1).

**Figure 2:**
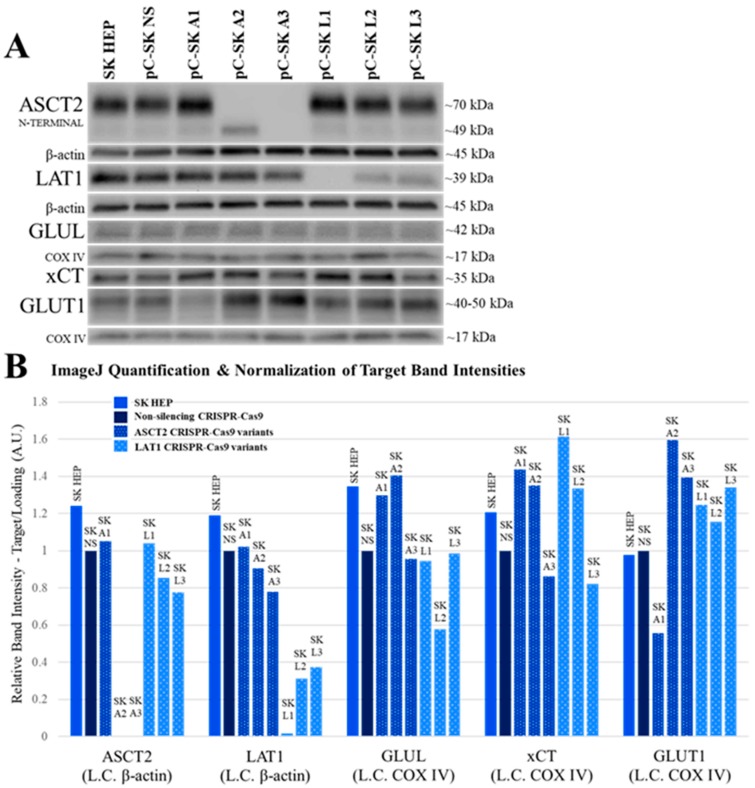
ASCT2, LAT1, GLUL, xCT, and GLUT1 protein in SKHep cell lines targeted for gene knockout with CRISPR-Cas9. Protein expression was measured by Western blot analysis as described in Materials and Methods. (**A**) The chemiluminescent bands for each target (ASCT2, LAT1, GLUL, xCT, and GLUT1) are shown in comparison to β-actin or COXIV protein detected on the same blots to control for loading error. One image for COXIV is shown for both xCT and GLUT1 because these proteins were detected on the same blot; (**B**) the band intensity of each target was quantified using ImageJ analysis software and normalized loading control (L.C.) band intensity in each lane.

In SKHep CRISPR-Cas9 cell lines, the ASCT2-targeted pC-SK A3 and the LAT1-targeted pC-SK L1 cell lines were the best knockouts of the ASCT2 and LAT1 proteins, respectively (Figure 2). Similar to HUH7, SKHep cells transfected with the A2 CRISPR-Cas9 plasmid construct also exhibited an unidentified, lower molecular weight band (Figure 2, ASCT2), although its size was larger than in HUH7 and seemed to represent an enhancement of a smaller (perhaps unglycosylated) species present in all other lines.

GLUL protein expression was not consistently affected by ASCT2 or LAT1 knockout (Figure 2, GLUL). The expression of xCT transporter protein was slightly enhanced (35–60% vs. NS) in the ASCT2-targeted pC-SK A1 and A2 cell lines as well as LAT1-targeted pC-SK L1 and L2 cell lines (Figure 2, xCT). Remarkably, GLUT1 transporter protein expression exhibited an inverse correlation with ASCT2 in SKHep cell lines. GLUT1 expression was diminished in the pC-SK A1 cell line where ASCT2 expression was oddly enhanced, whereas GLUT1 was increased (by 40–60% vs. NS) in pC-SK A2 and A3 cell lines, where ASCT2 was effectively knocked out (Figure 2, GLUT1).

Taken together, the results in both SKHep and HUH7 demonstrate that (1) expression of glutamine synthetase does not consistently change in response to ASCT2 knockout; (2) xCT transporter expression may be moderately responsive to ASCT2 and LAT1 knockout in SKHep; and (3) GLUT1 expression is enhanced in response to ASCT2 and LAT1 knockout only in SKHep-derived cell lines, but is reduced in HUH7. The only consistent pattern that emerged from the knockout studies is the inverse expression of GLUT1 vs. ASCT2 and in the mesenchymal SKHep lines.

Furthermore, ASCT2 knockout appears to be dependent on the CRISPR-Cas9 gRNA sequence identity and not dependent on cell line phenotype, based on the similar pattern of knockout effects observed in HUH7 and SKHep cell lines (Figure 1 vs. Figure 2). Conversely, the overall LAT1 knockout patterns between cell lines were different, and there may be cell line-specific characteristics of the LAT1 gene that favor the L3 CRISPR-Cas9 gRNA sequence in HUH7 and the L1 CRISPR-Cas9 gRNA sequence in SKHep.

### 2.4. CRISPR-Cas9 Markedly Represses Glutamine and Leucine Initial Rate Transport Activity in Epithelial (HUH7) and Mesenchymal (SKHep) Liver Cancer Cells

**Figure 3:**
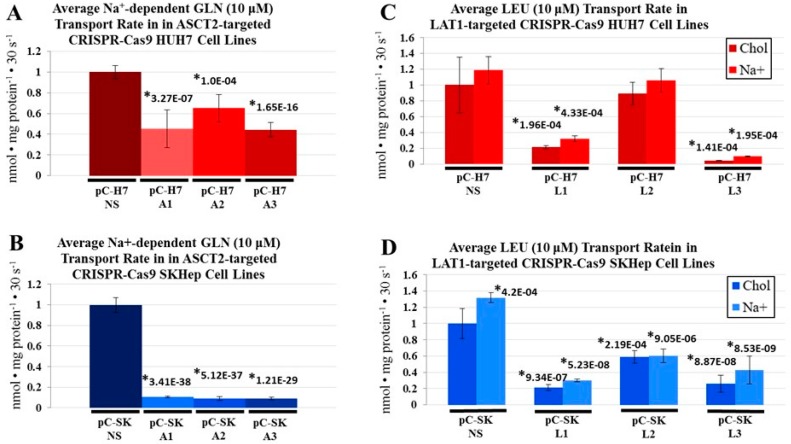
Na^+^-dependent glutamine (l-[^3^H]Gln) and leucine (l-[^3^H]Leu) initial rate uptake in CRISPR-Cas9 HUH7 and SKHep cell lines. Transport of 10 μM l-glutamine and 10 μM l-leucine was measured as described in Materials and Methods. Due to the Na^+^-dependence of ASCT2-mediated glutamine uptake, the transport values obtained in the absence of extracellular Na^+^ (diffusion and Na^+^-independent uptake) were subtracted from those in the presence of Na^+^ (total uptake) to yield Na^+^-dependent glutamine uptake rates depicted in graphs (**A**,**B**). For leucine transport, which is largely Na^+^-independent, transport rates in the absence (chol) and presence of Na^+^ are shown (**C**,**D**). To compare transport values between cluster plates, NS controls for each parent line were set to a value of 1 and all other transport values were mathematically adjusted to reflect this change. Data are the average of at least four separate determinations ± SD. Asterisks (*) denote values that are statistically significant at *p* < 0.050 compared to the same values in the NS control.

Initial rate transport analysis was performed using ^3^H-radiolabeled l-glutamine and l-leucine to evaluate the impact of ASCT2 and LAT1 transporter knockout on transporter activity (Figure 3). 

Similar to results from shRNA, the CRISPR-Cas9 system more robustly repressed ASCT2 transporter expression in the mesenchymal cell line (SKHep) than in the epithelial cell lines (Hep3B and HUH7). The most effective HUH7-derived ASCT2 knockout, pC-H7 A3, demonstrated a 56% reduction in Na^+^-dependent initial rate glutamine uptake (Figure 3A), and the best SKHep-derived ASCT2 knockout, pC-SK A3, demonstrated 91% reduction (Figure 3B), compared to nonsilencing controls (pC-H7 NS and pC-SK NS, respectively). The best LAT1 knockout derived from HUH7, pC-H7 L3, demonstrated a 96% reduction in initial rate leucine uptake (Figure 3C), and the best LAT1 knockout derived from SKHep, pC-SK L1, demonstrated 79% reduction (Figure 3D), compared to nonsilenced control cell lines (pC-H7 NS and pC-SK NS, respectively).

**Figure 4:**
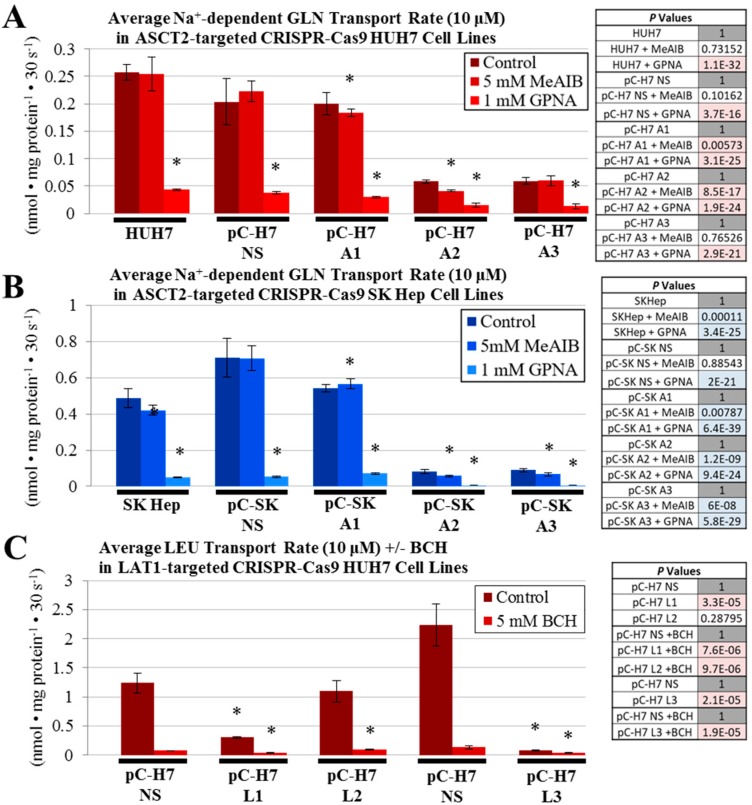

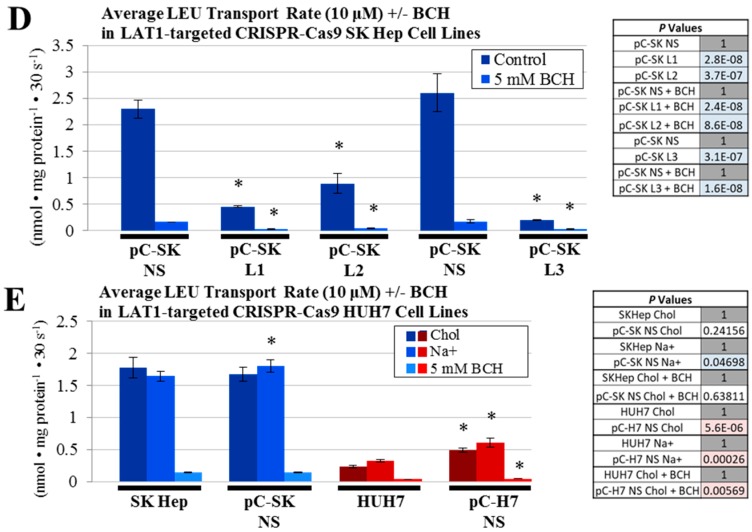
Na^+^-dependent glutamine (l-[^3^H]Gln) and Na^+^-independent leucine (l-[^3^H]Leu) initial rate uptake in the presence of transport inhibitors CRISPR-Cas9 HUH7 and SK Hep cell lines. Transport of 10 μM l-glutamine and 10 μM l-leucine was measured as described in Materials and Methods. Glutamine uptake mixes contained either MeAIB, a System A (SNAT1/SNAT2) inhibitor, or GPNA, a pan-specific glutamine transport inhibitor (**A**,**B**), and leucine uptake mixes contained BCH, a System L inhibitor (**C**–**E**). Leucine transport in control cell lines (**E**) was performed in Na^+^-containing and Na^+^-free (choline) buffers, as in Figure 3, while C and D were performed in Na^+^-free (choline) buffers. Data are the average of at least four separate determinations ± SD. Asterisks (*) denote values that are statistically significant with a *p* value < 0.050 compared to the respective control, as indicated in the adjacent tables.

To distinguish between glutamine transporters in control and ASCT2 knockout cells, two chemical inhibitors were alternatively added to the glutamine uptake mixes: (1) α-(methylamino)isobutyric acid (MeAIB), a System A (SNAT1/SNAT2) inhibitor and (2) l-γ-glutamyl-p-nitroanilide (GPNA), a pan-specific glutamine transport (e.g., ASCT2) inhibitor (Figure 4A,B). Na^+^-dependent glutamine uptake in the HUH7 ASCT2 knockout line pC-H7 A3 was not affected by treatment with MeAIB, yet GPNA reduced the already diminished glutamine uptake further, to 6% of the pC-H7 NS control (Figure 4A). In contrast, the other HUH7 ASCT2 knockout line pC-H7 A2 was sensitive to both MeAIB and more dramatically, GPNA. For the SKHep ASCT2 knockout pC-SK A2 and pC-SK A3 cell lines, the 10% residual initial rate glutamine uptake was significantly reduced by both MeAIB and more effectively, GPNA (Figure 4B). These results suggest that the 10–20% residual Na^+^-dependent glutamine transport activity in HCC ASCT2 knockout cells is not likely entirely attributable to SNAT1/2, a conclusion partially reinforced by lack of measurable upregulation of the SNAT2 System A transporter secondary to shRNA-mediated ASCT2 suppression (Supplemental Figure S14).

Likewise, initial rate leucine uptake was further characterized in LAT1 knockout lines, using the pan-specific System L-like transport inhibitor, 2-aminobicyclo-(2,2,1)-heptane-2-carboxylic acid (BCH), (Figure 4C–E). The residual initial rate leucine uptake maintained by all LAT1-targeted cell lines was reduced to near zero by BCH inhibition, similar to GPNA treatment in the ASCT2 knockout cells, suggesting that the residual activity post-knockout in both HUH7 and SKHep is likely mediated by other SLC7 (e.g., LAT2 or y^+^LAT) or SLC43 (LAT3 or 4) transporters. 

An additional comparison of initial rate leucine uptake was performed between controls, the parent cell lines versus their respective nonsilenced puromycin-resistant (NS) lines, in the absence or presence of Na^+^ (Figure 4E). There were no significant differences between these paired controls, except for a slight enhancement of leucine uptake in the HUH7 NS control compared to parent HUH7. As LAT1-mediated leucine transport activity is typically measured in Na^+^-free choline buffer, these data further indicated that a Na^+^-dependent leucine transporter did not compensate for the loss of LAT1.

Collectively, the results indicate that ASCT2 and LAT1 knockout in SKHep and HUH7 cell lines do not result in compensatory restoration of initial rate uptake by other glutamine and leucine transporters.

### 2.5. CRISPR-Cas9 ASCT2 and LAT1 Knockout Fails to Sustainably Repress Cell Growth in Epithelial (HUH7) or Mesenchymal (SKHep) Liver Cancer Cells

**Figure 5:**
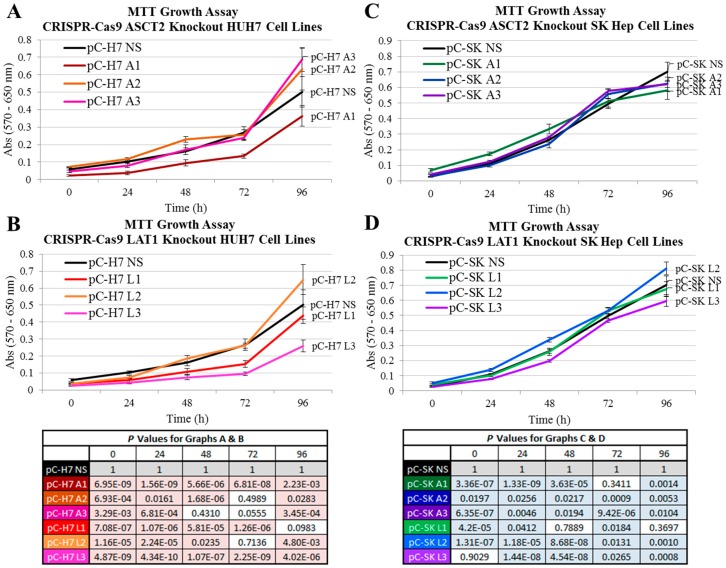
Growth in the ASCT2- and LAT1-targeted CRISPR-Cas9 HUH7 and SKHep cell lines. Growth was measured using an MTT assay as described in Materials and Methods. (**A**) Growth of HUH7 ASCT2-targeted cell lines (pC-H7 A1, A2, and A3) and (**B**) LAT1-targeted cell lines (pC-H7 L1, L2, and L3) are shown respective to the nonsilencing control cell line (pC-H7 NS). (**C**) Growth of SK Hep ASCT2-targeted cell lines (pC-SK A1, A2, and A3) and (**D**) LAT1-targeted cell lines (pC-SK L1, L2, and L3) are shown respective to the nonsilencing control cell line (pC-H7 NS). Colors in the tables match to cognate lines in the graph to facilitate reference; shaded values = significance at *p* < 0.050.

Colorimetric MTT analyses were performed to evaluate proliferation rates in the transporter knockout cell lines. In HUH7, the ASCT2-targeted pC-H7 A1 cell line grew at a moderately slower rate relative to the NS control (Figure 5A). However this cell line was also an unsuccessful ASCT2 knockout as determined by Western blot analysis (Figure 1), and the two successful ASCT2 knockout lines A2 and A3 grew slightly faster than NS, so reduced proliferation could not be attributed to diminished ASCT2 transporter expression. The two HUH7 LAT1 knockout lines that demonstrated the greatest reductions in initial rate leucine uptake, pC-H7 L1 and L3, also grew at a slower rate (Figure 5B), proportional to their degree of transporter repression (Figure 1 and Figure 3). In SKHep, there was no substantial difference in proliferation rates between transporter knockout and NS controls (Figure 5C,D). These results indicate that LAT1 knockout in HUH7 but not SKHep, led to statistically significant growth reductions, but that ASCT2 knockout failed to affect growth in either HCC cell type. However, all of the CRISPR-Cas9 transporter knockout cell lines were easily maintained long-term cell culture, suggesting that both epithelial and mesenchymal human HCC lines adapt to ASCT2 and LAT1 knockout.

### 2.6. CRISPR-Cas9 ASCT2 and LAT1 Knockout Does Not Affect mTORC1 Signaling in Epithelial (HUH7) or Mesenchymal (SKHep) Liver Cancer Cells

Similar to the studies in the shRNA suppressed lines, the intensity of mTORC1-mediated growth signaling in the transporter knockout cell lines was assessed by Western blot analysis to measure the phosphorylation state 4E-BP1, a direct target of mTORC1 serine threonine kinase activity. Assessment of mTORC1 activity was implemented under controlled conditions of growth factor stimulation, amino acid availability, and rapamycin treatment. Overall, neither the HUH7 nor the SKHep CRISPR-Cas9 transporter knockout cell lines exhibited any qualitative differences in 4E-BP1 phosphorylation state relative to their respective nonsilencing control cell lines (Supplemental Figures S22 and S23). Simultaneous removal of serum and amino acids provoked the most pronounced suppression of higher level 4E-BP1 phosphorylation (an index of mTORC1 activity) in control and knockout cell lines uniformly.

### 2.7. CRISPR-Cas9 ASCT2 and LAT1 Knockout Does Not Alter Glutamine and Leucine Intracellular Accumulation or Incorporation into Macromolecules

The lack of impact on cell growth, viability, or mTORC1 signaling in the face of demonstrated ASCT2 or LAT1 knockout, as well as the absence of any measurable or obvious compensatory responses to dramatic reductions in glutamine and leucine transport, were surprising and unexpected. One potential explanation for these results was the possibility that acute reductions in initial-rate amino acid transport did not translate to longer-term, steady-state reductions in intracellular amino acid economies. To test this possibility, prolonged uptake of radiolabeled l-glutamine and l-leucine was performed for an incubation time of 60 min (Supplemental Figure S24). It is important to note that, unlike the earlier initial rate uptake experiments, these amino acid accumulation experiments were performed in the presence of normal media concentrations of all amino acids, serum, glucose, and sodium bicarbonate. Therefore, these measurements are reflective of total glutamine or leucine uptake under normal growth conditions in vitro instead of the austere Krebs–Ringer buffer used in initial-rate transport measurement.

After 60 min, the LAT1 knockout pC-H7 L3 and pC-SK L1 cell lines, derived from HUH7 and SKHep, respectively, exhibited small (10–15%) increases in glutamine accumulation (Supplemental Figure S24A,B). Additionally, the ASCT2 knockout HUH7 cell line, pC-H7 A3, showed a modest (10%) reduction in glutamine accumulation (Supplemental Figure S24A), and the LAT1 knockout SKHep cell line, pC-SK L1, also yielded a moderate (20%) reduction in leucine accumulation (Supplemental Figure S24D). Strikingly, these differences in accumulation were small in scale compared to the much more extensive 60–90% reductions in glutamine and leucine initial rate uptake between the knockout and NS control cell lines (Figure 3). Despite transporter knockout in these cell lines, the results suggest that the cells remain capable of accruing similar relative intracellular concentrations of amino acids over time, which may explain why transporter knockout cell lines fail to exhibit a significantly impaired growth phenotype or mTORC1 signaling despite substantially decreased initial-rate glutamine and leucine uptake.

**Figure 6:**
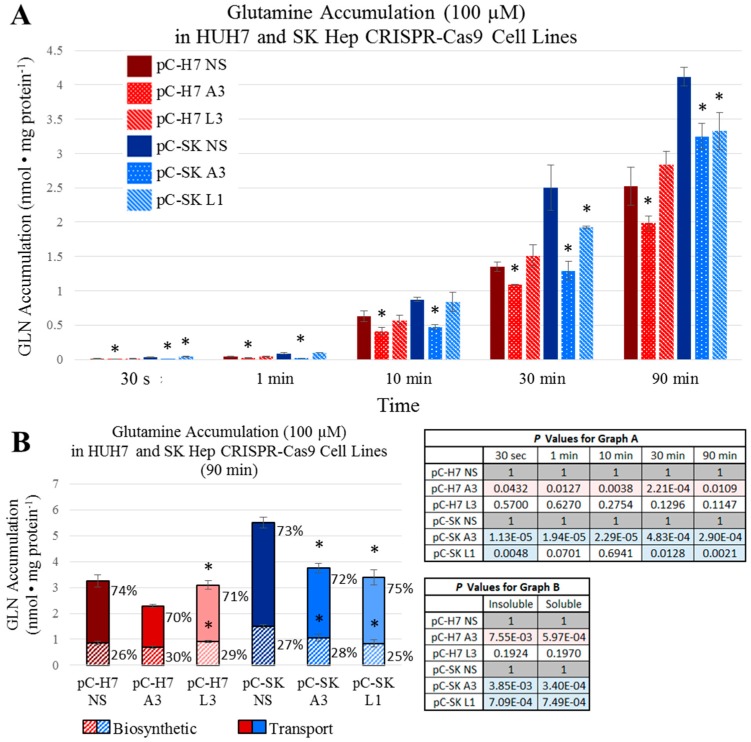
Intracellular glutamine (l-[^3^H]Gln) accumulation over time. Accumulation of 100 μM l-glutamine was measured as described in Materials and Methods. (**A**) Intracellular accumulation of glutamine was measured in ASCT2- and LAT1-targeted HUH7 cell lines (pC-H7 A3 and L3, respectively) compared to the nonsilencing control cell line (pC-H7 NS); and in ASCT2- and LAT1-targeted SK Hep cell lines (pC-SK A3 and L1, respectively) compared to the nonsilencing control cell line (pC-SK NS); (**B**) Intracellular fractions of free (Transport) and biosynthetically incorporated (Biosynthetic) radioactive l-glutamine were measured after 90 min of accumulation as described in Materials and Methods. Data are the average of at least four separate determinations ± SD. Asterisks (*) denote values that are statistically significant at *p* < 0.050 vs. NS control; actual p values are shown in the adjacent tables with pink or blue shaded values indicating *p* < 0.050.

**Figure 7:**
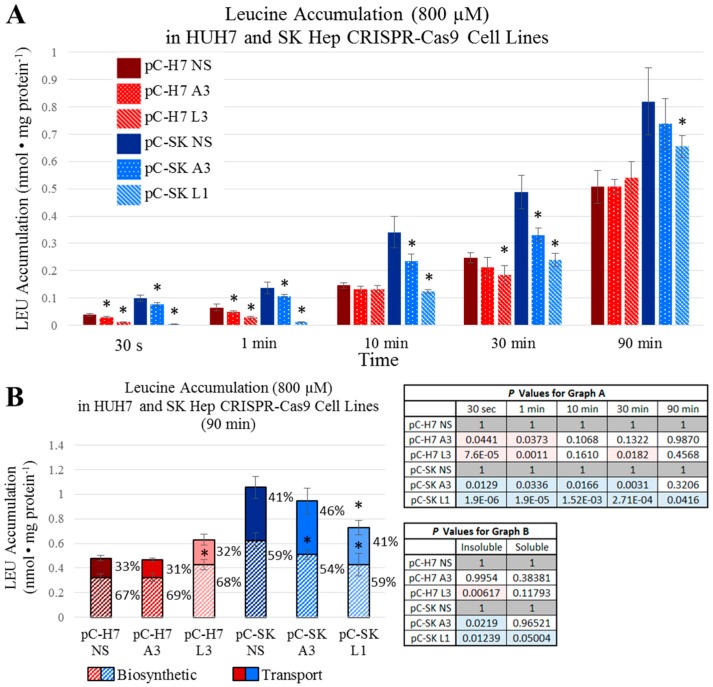
Intracellular leucine (l-[^3^H]Leu) accumulation over time. Accumulation of 800 μM l-leucine was measured as described in Materials and Methods. (**A**) Intracellular accumulation of leucine was measured in ASCT2- and LAT1-targeted HUH7 cell lines (pC-H7 A3 and L3, respectively) in comparison to the nonsilencing control cell line (pC-H7 NS), and in ASCT2- and LAT1-targeted SK Hep cell lines (pC-SK A3 and L1, respectively) in comparison to the nonsilencing control cell line (pC-SK NS); (**B**) Intracellular fractions of free and biosynthetically incorporated radioactive l-leucine were measured after 90 minutes of accumulation as described in Materials and Methods. Data are the average of at least four separate determinations ± SD. Asterisks (*) denote values statistically significant at *p* < 0.050 vs. NS control; actual p values are shown in the adjacent tables with pink or blue shaded values indicating *p* < 0.050.

To assess how rapidly the ASCT2 and LAT1 knockout cell lines are able to restore intracellular concentrations of glutamine and leucine comparable to controls, uptake analysis was performed with ^3^H-radiolabeled l-glutamine and l-leucine at specific time increments over the span of 90 min (Figure 6 and Figure 7). For glutamine, in the HUH7 ASCT2 knockout pC-H7 A3, demonstrated significantly reduced intracellular accumulation at all time points (Figure 6A). However, this difference became less substantial over time, starting at a 41% initial difference at 30 s and ending in a 21% difference after one and half hours. In SKHep, the ASCT2 knockout pC-SK A3 was significantly different from the nonsilencing pC-SK NS control cell line at all time points for intracellular glutamine accumulation. However, these differences became considerably smaller over time, and changed from an initial 90% difference at 30 s to a 21% difference after 90 min (Figure 6A). As suspected, marked initial disparities in glutamine uptake in ASCT2 knockout cells waned over time in both HUH7 and SKHep.

For leucine, the SKHep LAT1 knockout line pC-SK L1 demonstrated an initial 96% decrease in leucine accumulation vs. NS controls at 30 s, which reduced to a 20% difference after 90 min (Figure 7A). The HUH7 LAT1 knockout line pC-H7 L3 demonstrated a large reduction in intracellular leucine accumulation at 30 s and 1 min (70% and 56%, respectively), but this difference was reduced to nonsignificant levels by 10 min (Figure 7A). These results suggest that both epithelial and mesenchymal HCC cell lines are able to maintain intracellular concentrations of glutamine and leucine comparable to controls in the face of ASCT2 and LAT1 knockout, and that profound reductions in initial rate uptake by these exchangers do not translate to significantly depressed intracellular amino acid availability in vitro.

Finally, to determine whether ASCT2 or LAT1 knockout affects rates of glutamine or leucine incorporation into macromolecules (e.g., charged transfer RNA, proteins, and nucleic acids), cells were incubated in ^3^H-radiolabeled l-glutamine and l-leucine for 90 min followed by precipitation with ice-cold 10% trichloroacetic acid (TCA). The intracellular ratio of free (acid-soluble; labeled in figures as ‘Transport’) to incorporated (acid-precipitable; labeled as ‘Biosynthetic’, in figures) glutamine and leucine was calculated (Figure 6B and Figure 7B, respectively). Amino acid disposition was not radically different across any of the ASCT2 or LAT1 knockout cell lines, and this ratio ranged between 70:30% and 75:25% (free:incorporated) for glutamine (Figure 6B). Likewise, the intracellular ratio of free to incorporated leucine was not significantly different within the HUH7 and SKHep sets of CRISPR-Cas9 cell lines (Figure 7B). In general, there was a greater fraction of free leucine in SKHep compared to HUH7 by roughly 10% of the total intracellular pool. For leucine in HUH7, the ratios ranged between 31:69% and 33:67%, and for SKHep, the ratio ranged between 41:59% and 46:54%. These results indicate that transporter knockout of ASCT2 or LAT1 does not affect the percentage of transported amino acid that is biosynthetically utilized over time. 

### 2.8. Revisiting Prior Investigations with Antisense RNA

A recurrent problem in this current body of work has been the incomplete silencing of ASCT2 and LAT1. Even when Western blots fail to detect target transporter protein in shRNA- and CRISPR-Cas9-targeted cell lines, the possibility remains that the residual activity remaining secondary to ASCT2 or LAT1 knockout (e.g., Figure 1, Figure 2, Figure 3 and Figure 4) might be vestigial remnants of these two transporters.

**Figure 8:**
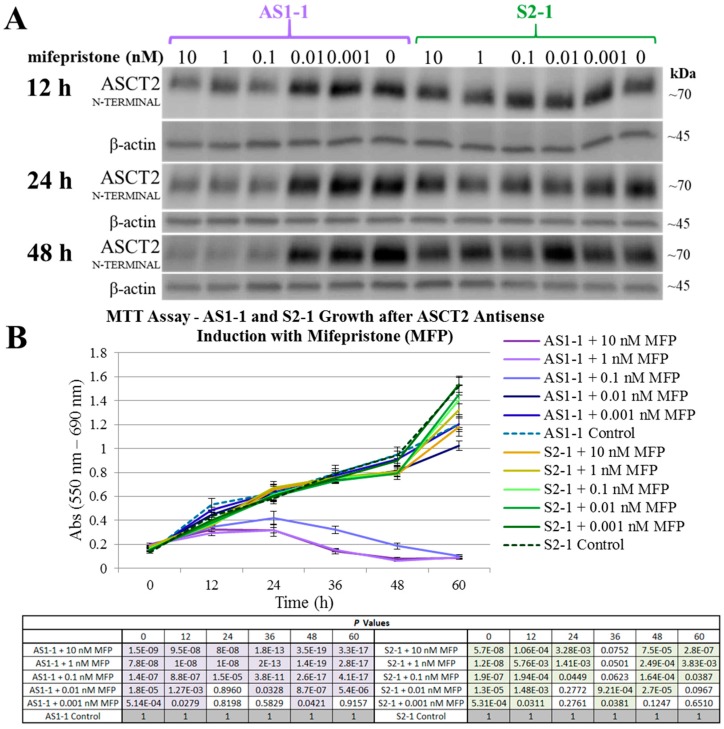
Mifepristone (MFP) tonic regulation of ASCT2 protein expression and growth in AS1-1 and S2-1 SKHep-derived cell lines. AS1-1 and S2-1 are MFP-inducible antisense and sense ASCT2 RNA cell lines established by our lab as an early system for studying ASCT2 silencing in SKHep [31]. Inducible expression of a long 1.3 kb ASCT2 antisense RNA in the AS1-1 cell line elicits a well-characterized apoptotic response, and the sense S2-1 cell line serves as a control in which no antisense RNA is generated. Here, these two cell lines were treated with a serial dilution of MFP concentrations (10 pM–10 nM), and ASCT2 protein expression and growth over time was measured by Western blot and MTT analysis, respectively, both as described in Materials and Methods. (**A**) The chemiluminescent bands for ASCT2 for each time point are shown compared to β-actin detected on the same blots to control for loading; (**B**) MTT analysis of cell growth secondary to MFP treatment; AS1-1 growth quantification is shown in shades of purple and control S2-1 cell line in shades of green. The statistical *p*-values for MTT absorbance values between MFP treatments and cognate controls for each line (AS1-1 and S2-1) are shown in the table below the graph, with those reaching *p* < 0.050 vs. control shaded in purple or green. Asterisks denoting statistically significant differences between treated and control values have been omitted for clarity.

To address the possibility that even 10% of the original activity of ASCT2 might be sufficient to sustain growth, a system previously developed in our lab was deployed: the inducible antisense cell lines originally derived from SKHep—antisense (AS1-1) and control sense (S2-1) cells [31]. Tonic control of the system was achieved by serial dilutions of the mifepristone (MFP) inducing agent. After AS1-1 and S2-1 cells were treated with serial dilutions of MFP, ASCT2 protein expression was measured by Western blot analysis (Figure 8A) and cell growth was measured over time by MTT assay (Figure 8B). The minimum concentration of MFP that resulted in cell death was 0.1 nM. Importantly, at the time of comprehensive apoptotic death (48 h) and at a dosage of MFP (10 nM) deployed in earlier studies, ASCT2 protein is still detectable by Western blot analysis at levels that exceed those achieved with both shRNA and CRISPR-Cas9 knockout (Figure 8A vs. Supplemental Figure S11 and Figure 2). 

These results indicate that the apoptotic cell death after ASCT2 knockdown in the antisense RNA system did not require a complete elimination of the ASCT2 transporter protein, and instead must be attributable to other factors independent of ASCT2 reduction. Therefore, the conclusions made by this early study can now be re-evaluated through the use of more contemporary methods of amino acid transporter targeting, the shRNAmir, and CRISPR-Cas9 silencing systems. The collective results of the current study indicate that ASCT2 and LAT1 are each not alone critical to support the survival and proliferation of human hepatoma cells in vitro.

## 3. Discussion

The studies presented here represent a culmination of several years of research identifying and pursuing amino acid transporters as potential targets for cancer therapy. ASCT2 and LAT1 are both transporters that have been implicated by our lab and others in driving the growth of cancerous cells. Prior studies from our lab implicated ASCT2 as vital for HCC cell survival [31] and mTORC1 signaling [15], and also, implicated LAT1 as a “partner in crime” in driving the growth of many human cancers [10]. The overarching question has been “can ASCT2 or LAT1 be effective targets for cancer therapy?” Likewise, the studies here were designed to assess whether either transporter might play a particularly important role in driving the biology of epithelial (modeling primary) and mesenchymal (modeling invasive/metastatic) cancer cells. The results suggest that alone, neither transporter is necessary for sustained in vitro growth of epithelial or mesenchymal human liver cancer cells.

Using more refined and contemporary approaches (shRNA and CRISPR-Cas9 systems) in the present study relative to our prior studies with inducible ASCT2 antisense RNA, both ASCT2 and LAT1 were successfully repressed or eliminated below detectable levels. The results suggest that cancer cells adapt to profound transporter suppression—likely far beyond what could be reasonably obtained in vivo. In spite of effective ASCT2 or LAT1 knockout (Figure 1 and Figure 2), and linked reductions in glutamine and leucine transport rates (Figure 3 and Figure 4), both epithelial and mesenchymal human HCC cells continued to grow at rates equal to, or only moderately less than cognate controls (Figures 5). These results were further reinforced by the observation that neither LAT1, nor ASCT2 CRISPR-Cas9 knockout cells exhibited sustained repression of intracellular glutamine or leucine accumulation, incorporation into protein and other macromolecules (Figure 6 and Figure 7), or reductions in long-term or acute mTORC1 signaling (Supplemental Figures S22 and S23). Collectively, all results were consistent—human liver cancer cells adapt to profoundly diminished ASCT2 or LAT1 activity.

The results in this study regarding ASCT2 also contradict earlier work from our laboratory using an inducible ASCT2 antisense RNA, which showed that ASCT2 silencing led to apoptotic cell death and early depression of mTORC1 signaling [15,31]. The reason for the disparity is likely grounded in off-target effects of the long ASCT2 antisense RNA deployed in that study [34]. Using CRISPR-Cas9, ASCT2 expression levels in HUH7 (Figure 1) and SKHep (Figure 2) were repressed to levels that far exceed those achieved with the antisense RNA targeting methodology (Figure 8). Therefore, the cause of cell death in prior work was likely not alone attributable to ASCT2 suppression. The induced antisense transcript was large, roughly ~1.3 kb in length, complementary to the endogenous ASCT2 mRNA transcript, but a Protein Kinase R (PKR)-induced stress response was ruled out as an off-target effect in those studies [31]. However, it is possible that once the antisense transcript formed a double-stranded complex with the endogenous ASCT2 mRNA transcript and is subsequently cleaved by Dicer, the resulting array of small microRNA (miRNA) duplexes could (1) allow the RNA-induced silencing complex (RISC) to target more than just the endogenous ASCT2 mRNA transcript, or (2) overwhelm the cellular miRNA processing machinery leading to cell death [35]. Therefore, ASCT2 may not have as critical of a role in hepatoma cell survival in vitro as previously thought, when evaluated in the light of results presented here.

Is all hope lost in targeting these transporters for cancer therapy based on these results? In a word, no. If anything, the results presented here further inform our approach to targeting metabolic derangements, including the so-called “glutamine addiction” of cancer cells [17]. Old models are constantly refined by often unexpected experimental results, and subsequent paradigm shifts. In this case, the old model that native levels of ASCT2 or LAT1 activity are necessary for cancer cell growth and mTORC1 signaling were not supported by our data. Recent studies have shown that tumorigenic potential is markedly diminished in ASCT2 knockout cancer cells [14,36]; likewise, the same was true in LAT1 knockout cells [30]. The ability to support in vitro growth and the attributes required for effective tumorigenesis (stromal cell recruitment, angiogenesis, establishment of tumor metabolic economies, etc.), while related, are likewise quite distinct. 

As cancer is now widely recognized as a disease of tissues, not just cells, stromal-carcinoma metabolic cycles clearly must be established in order for a tumor to form, grow, and progress. Recent work showed despite moderate to no effects on in vitro growth, ASCT2 knockout cells generate smaller tumors in mouse xenograft models [14,36]. We hypothesize that the disparity in lack of an in vitro growth phenotype and diminished tumorigenic potential resides in the compromised ability of the carcinoma cells to engage in metabolic cycling, which is crucial in tumors [37]. In other words, while loss of a rapid exchange mechanism (ASCT2 or LAT1) has modest effects if any, on in vitro growth and long-term amino acid accumulation (Figure 5, Figure 6 and Figure 7) in a nutrient-rich environment, in a tissue context where resources are more austere, the inability to sufficiently fuel metabolic cycles through export likely compromises the tissue-generating stromal recruitment and support component of tumorigenesis. 

A new model has emerged for the role of amino acid transporters in cancer: Both ASCT2 and LAT1 are obligate amino acid exchangers—a role characterized as cellular “harmonizers” by Bröer et al. [14], as they equilibrate intracellular amino acid levels to align with metabolic demands. Other Na^+^-dependent transporters known as “loaders” mediate net unidirectional delivery of amino acids, such as members of the SLC38 System N and A transporters [38]. While slower than harmonizers, they provide substrates that sustain mTORC1 activity either directly or indirectly, which is likely why we saw no effect of ASCT2 or LAT1 knockout on basal or amino acid-induced mTORC1 signaling, as reported and suggested by Bröer et al. [14]. To fulfill their physiological role, the harmonizers ASCT2 and LAT1 necessarily must have higher innate activities than loaders, and indeed this is precisely why they have been regarded as “dominant” glutamine transporters, especially when initial-rate amino acid transport is measured as an assessment of carrier contributions [11]. As harmonizers, ASCT2 and LAT1 provide substrates via exchange to surrounding cells (e.g., stromal cells), whose metabolic economies are likely complimentary to the carcinoma cells [37]. One cell’s byproduct is another cell’s fuel. Their export role in a heterotypic tumor is likely why the emerging studies to-date have indicated modest in vitro growth effects of LAT1 or ASCT2 knockout in different human cancer cell types, but more profound impacts on tumorigenesis [14,36]. Studies are currently underway to test whether the same holds true in the ASCT2 and LAT1 knockout SKHep and HUH7 liver cancer cell cohorts reported here.

Another facet of targeting amino acid transporters in cancer that will come to bear is the integrated (amino acid) stress response (ISR) [39,40]. In the ISR, amino acid starvation, as might be induced in vivo by transporter knockout, induces a general control nonderepressible 2 (GCN2) kinase-dependent phosphorylation of the translational regulator eukaryotic initiation factor 2 alpha (eIF2α) and arrest of cap-dependent translation, while activation of transcription factor ATF4 leads to expression of a third functional class of amino acid transporter—“rescue”—such as SNAT2 [39]. In recent work, Bröer et al. reported that upon ASCT2 knockout in HeLa and osteosarcoma cells, an ISR upregulated the “loader” SNAT1 to functionally replace ASCT2 in a GCN2-dependent manner [14]. In contrast, ASCT2 knockout in colon and lung carcinoma cells did not induce an ISR, whereas LAT1 knockout did so [14,36]. While the ISR was not investigated extensively in the studies reported here, no evidence of compensatory SNAT1 or SNAT2 upregulation was observed, but ongoing studies are assessing these links more deeply. Clearly the ISR (amino acid—GCN2/eIF2α/ATF4 stress response) will be a key consideration in targeting amino acid transporters in oncology, especially as it relates to mechanisms of developed resistance. 

One last possibility exists in the targeting of ASCT2 and LAT1 in cancer therapy: double-knockout. No one has yet reported the double knockout of ASCT2 and LAT1, which may be lethal to the cells. Attempts by our lab to generate double-knockouts were unsuccessful, which suggests, but certainly does not prove, that an ASCT2^−/−^/LAT1^−/−^ genotype might be lethal to cancer cells. We are currently exploring this possibility using different approaches.

In conclusion, the studies presented here indicate that ASCT2 and LAT1 can each be successfully targeted and eliminated, but neither knockout alone is sufficient to prevent cancer cell growth in vitro, contrary to earlier RNAi-based studies that suggested critical roles for each. Such disparities are not uncommon and continue to emerge in oncology [32]. Recent studies demonstrating that ASCT2 or LAT1 knockout diminishes human cancer cell tumorigenesis in mouse xenograft models likewise indicates that their role as amino acid exchangers is likely more essential in vivo than in vitro. The field of amino acid transporters as therapeutic targets still holds promise in this regard, informed now by recent research results and linked paradigms for experimental design.

## 4. Materials and Methods

### 4.1. Cell Culture

The human hepatoma cell lines used herein were Hep3B, HUH7, and SKHep cells (American Type Culture Collection, Manassas, VA, USA). Two additional cell lines, AS1-1 and S2-1, were used that were previously established by transfecting SKHep with an inducible, dual vector system which produces an antisense RNA against the ASCT2 mRNA transcript, as previously described [31]. All cell lines were maintained at 37 °C in a humidified atmosphere of 5% CO_2_-95% air in Dulbecco’s Modified Eagle Medium (DMEM, 4.5 mg/mL d-glucose) (Gibco, Waltham, MA, USA) supplemented with 10% triple 0.1 μm filtered fetal bovine serum (FBS) (Atlanta Biologicals, Flowery Branch, GA, USA), 2 mM l-glutamine (Thermo Scientific, Waltham, MA, USA), 1% antibiotic/anti-mycotic solution (100 U/mL penicillin G, 100 μg/mL streptomycin, and 0.25 μg/mL amphotericin; Thermo Scientific). All cell lines were authenticated by short tandem repeat (STR) analysis by the Johns Hopkins Genetics Resource Core Facility (Baltimore, MD, USA). Cell count measurements were performed using an Invitrogen™ Tali™ image-based cytometer (Thermo Scientific) and a hemocytometer (Hausser Scientific Co., Horsham, PA, USA).

### 4.2. RNAi—shRNAmir Transfection and Antibiotic Selection

pGIPZ plasmid vectors were obtained from Open Biosystems as *Escherichia coli* (*E. coli*) stocks (60% glycerol) and stored at −80 °C [41]; pGIPZ plasmid constituents are depicted in Supplemental Figures S3 and S4, respectively. Five shRNAmir sequences were used to target the alanine-serine-cysteine transporter 2 (ASCT2) mRNA transcript (clone IDs: V3LHS_393625, V3LHS_393624, V3LHS_393623, and V3LHS_393622, and V2LHS_56725), and these shRNAmir designs are referred to herein as A1, A2, A3, A4, and A5, respectively. Four shRNAmir sequences selected to target the large neutral amino acid transporter 1 (LAT1) mRNA transcript (clone IDs: V2LHS_17487, V3LHS_353675, V3LHS_353676, and V3LHS_353679), and these shRNAmir designs are referred to herein as L1, L2, L3, and L4, respectively. The glyceraldehyde-3-phosphate dehydrogenase (GAPDH) shRNA transcript used as a positive GIPZ system control is V3LHS_382663. The scrambled shRNAmir sequence that served as a nonsilencing (NS) negative control is RHS4346. The binding sites for each shRNA variant on the target ASCT2 and LAT1 mRNA sequences (accession numbers NM_005628 and NM_003486, respectively) were visualized using Sfold Software [42,43] (Supplemental Figures S5 and S6). The shRNAmir sense and anti-sense sequences are listed in Supplemental Table S1. All pGIPZ shRNAmir plasmid vectors.

PureYield™ Promega plasmid midiprep kit (Promega, Madison, WI, USA) was used to extract and purify plasmid stocks according to the manufacturer protocol. Plasmid concentrations were measured with a Nanodrop 2000 Micro-Volume UV-Vis spectrophotometer (Thermo Scientific), and each plasmid stock was stored at −20 °C. Transfections were performed by electroporation into suspended HCC cells (5 × 10^6^ cells/mL) using the Neon^®^ Transfection System (Thermo Scientific). All stably transfected hepatoma cells were maintained under selective pressure in growth media supplemented with 0.5 μg/mL puromycin (Gibco) for SKHep and 3.0 μg/mL for Hep3B; antibiotic concentrations were established by preliminary survival dosing assays. Expression of the GIPZ vector’s visual reporter, turboGFP (tGFP), was imaged using an EVOS^®^ FLoid^®^ fluorescent microscope (Invitrogen, Carlsbad, CA, USA) with an 482/18 nm excitation preset (Supplemental Figures S7 and S8).

### 4.3. CRISPR-Cas9 Transfection and Antibiotic Selection

pCLIP-All-EFS-Puro CRISPR-Cas9 all-in-one plasmid vectors were obtained from transOMIC technologies as PrimePlus *E. coli* stocks (60% glycerol) and stored at −80 °C [44]. A comprehensive map of the pCLIP-All-EFS-Puro vector plasmid vector is depicted in Supplemental Figure S21. Three CRISPR-Cas9 sequences were selected to target the *SLC1A5* gene (clone IDs: TEVH-1126600, TEVH-1193742, and TEVH-120884); these sequences are referred to herein as A1, A2, and A3, respectively. Three CRISPR-Cas9 sequences were selected to target *SLC7A5* gene (clone IDs: TEVH-1112259, TEVH-1179401, and TEVH-1246543), and these sequences referred to herein as L1, L2, and L3, respectively. The clone ID of the non-silencing CRISPR-Cas9 sequence that served as a CRISPR-Cas9 silencing system control is TELA1011, referred to herein as NS. Each unique guide RNA (gRNA) sequence is listed in Supplemental Table S2, and the targeted regions in each gene were determined using the following accession numbers: NC_000019.10 and NC_000016.10 for *SLC1A5* and *SLC7A5*, respectively. pCLIP-All-EFS-Puro plasmid preparation, quantification, electroporation, and selection procedures were the same as described earlier for the pGIPZ shRNA plasmid vectors, with the exception that the HUH7 cell line was used instead of Hep3B. Stably transfected cells were maintained under selective pressure in growth media supplemented with 0.5 μg/mL puromycin (Gibco) for SKHep and 3.0 μg/mL for HUH7.

### 4.4. RNA Isolation and RT-qPCR

All cell lines were cultured in 150 mm culture dish; when the cells reached 80–90% confluence, an RNA isolation was performed using TRIzol^®^ reagent (Invitrogen, Carlsbad, CA, USA) according to the manufacturer instructions, and this was followed by an alcohol precipitation to concentrate the samples. RNA integrity was assessed by visualization of 28S and 18S band quality after electrophoresis on a 1% agarose gel. A Nanodrop 2000 Micro-Volume UV-Vis spectrophotometer was used to quantify RNA concentrations. All concentrated RNA samples were stored at −80 °C until use. One µg of total RNA was used from each sample for reverse transcription (RT) reactions, which contained reverse transcriptase, oligo(dT), 5× reaction buffer, MgCl_2_, RNasin, dNTP (all from Promega). RT-qPCR was performed using proprietary TaqMan^®^ Gene Expression Assay primers exclusive to human ASCT2, LAT1, alanine-serine-cysteine transporter 1 (ASCT1), sodium-dependent neutral amino acid transporter 2 (SNAT2), large neutral amino acid transporter 2 (LAT2), glutamine synthetase (GLUL), and cystine-glutamate transporter (xCT) mRNA transcripts, and all measurements were normalized to two housekeeping genes (HKG): TATA-binding protein (TBP) and hydroxymethylbilane synthase (HMBS) (all TaqMan probes were from Applied Biosystems, Foster City, CA, USA). All primer sets selected for this study spanned intragenic regions (introns) within each gene. The 2^−ΔΔ*C*t^ method was used to calculate the fold difference in expression relative to nonsilencing (NS) controls generated from each parent cell line. Greater than a two-fold increase in expression was considered significant. All RT-qPCR reactions were carried out using Eppendorf Mastercycler^®^ ep Realplex instrumentation (Eppendorf, Hauppauge, NY, USA) and analytical software (Eppendorf Realplex software, Version 2.2). Each run was performed a minimum of three times, and within each run, each sample was run in triplicate for each target transcript. These experiments complied with MIQE (Minimum Information about Quantitative Real-Time PCR Experiments) guidelines [45].

### 4.5. Western Blot Analysis

For Western blot analysis of ASCT2, LAT1, GAPDH, GLUL, and glucose transporter 1 (GLUT1), total cellular protein lysates were prepared using 2× Laemmli lysis buffer (2% SDS (Sigma, St. Louis, MO, USA), 62.5 mM Tris-HCl (pH 6.8), and 1% protease inhibitor cocktail (Thermo Scientific)). Lysis was performed in 6-well plates when the cell monolayers reached confluence. Total cellular protein concentration was measured with a Nanodrop 2000 Micro-Volume UV-Vis spectrophotometer, and equal protein from each sample was prepared for vertical electrophoresis in 2% SDS, 10% glycerol, 62.5 mM Tris-HCl (pH 6.8), 0.05% bromophenol blue, and 50 mM dithiothreitol (DTT) (Fisher Scientific). For Western blot analysis of mTORC1 phosphorylated targets (4EBP1 and p70S6K), total cellular protein lysates were prepared using a lysis buffer which contained phosphatase inhibitors (10 mM Tris (pH 6.8), 1 mM EDTA, 5 mM EGTA, 0.5% NP40 (US Biological, Salem, MA, USA), 0.1% TritonX 100, 1 mM sodium orthovanadate (Sigma), 50 mM β-glycerophosphate (Sigma), and 1% protease inhibitor cocktail (Thermo Scientific)). This lysis buffer formulation was used for the analysis of total and phosphorylated eukaryotic initiation factor 4E-binding protein 1 (4E-BP1^TOTAL^ and 4E-BP1^T37/46^) and 70 kDa ribosomal protein S6 kinase (p70S6K^TOTAL^ and p70S6K^T389^). Total cellular protein concentration was measured with a bicinchoninic acid (BCA) chemical assay (Pierce Chemical, Rockford, IL, USA) and an Epoch microplate spectrophotometer. Equal protein from each sample was prepared for vertical electrophoresis in the same buffer that was used for lysis, supplemented with 10% glycerol, 0.05% bromophenol blue, and 50 mM dithiothreitol (DTT). All of the total cellular protein lysates analyzed in 4E-BP1 and p70S6K Western blots were run in parallel with a positive control cell lysate from MCF7 cells treated with insulin (Cell Signaling Technologies, Danvers, MA, USA).

For all Western blots, vertical electrophoresis was run on precast 4–20% Mini-PROTEAN^®^ TGX™ polyacrylamide gels (Bio-Rad, Hercules, CA, USA) at 150 V for 45 min, and protein samples were transferred to Immobilon-P polyvinylidene difluoride (PVDF) membranes (Millipore) at 75 V for 90 min. After 1 h of incubation in blocking buffer (5% bovine serum albumin (BSA) in TBST (0.1% Tween-20, 150 mM NaCl, 20 mM Tris (pH 7.5)) at room temperature, the PVDF membranes were incubated overnight at 4 °C with the appropriate primary antibody in blocking buffer. After four washes with TBST), membranes were incubated with secondary antibody in blocking buffer for 1 h at room temperature, and immunoreactive bands were visualized by adding 10 mL of chemiluminescent substrate (LumiGLO^®^ and peroxide both from Cell Signaling Technologies) and detected with a G:Box chemiluminescence imager (Syngene, Frederick, MD, USA) and GeneSnap image acquisition software (Syngene, version 7.09). The molecular size of the chemiluminescent bands was determined by comparison to a biotinylated ladder (Cell Signaling Technologies), and band intensities were quantified using ImageJ software (Available online: https://imagej.nih.gov/ij/download.html). Target band intensity was normalized to the band intensities of the loading controls measured on the same blot. For Western blots that measured total and phosphorylated 4E-BP1, a change in kDa weight of the 4E-BP1 protein due to phosphorylation was the principal focus, and banding patterns for 4E-BP1 Western blots were qualitatively evaluated.

Primary polyclonal rabbit IgG antibodies against LAT1 were obtained from Abcam (Cambridge, MA, USA). Primary IgG antibodies against GLUT1 and xCT were obtained from Thermo Scientific. All other primary monoclonal rabbit IgG antibodies as well as secondary HRP-conjugated goat, anti-rabbit IgG antibodies were obtained from Cell Signaling Technologies. Manufacturer-recommended antibody concentrations were applied to each blot during all hybridization steps.

### 4.6. Glutamine and Leucine Transport Analysis

All cell lines were seeded at a density of 1 × 10^5^ cells/well in 24-well culture plates and allowed to grow to 80–90% confluence, normally one or two days later. Radiolabeled glutamine (l-[^3^H]Gln) and leucine (l-[^3^H]Leu) (Perkin-Elmer, Waltham, MA, USA), each at a 3 μCi/mL were used to assess amino acid transport as described previously [11,31]. Initial-rate transport measurements were performed in Na^+^-containing (NaKRP) or Na^+^-free (cholKRP) Krebs–Ringer Phosphate Buffers in the presence of unlabeled l-Gln or l-Leu, each at 10 μM—herein referred to as “uptake mix”. All transport measurements were carried out at 37 °C and were terminated after 30 s by three rapid washes with an ice-cold phosphate-buffered saline (PBS) solution. Transported amino acids were extracted with lysis buffer (0.2 mL/well of 0.2% SDS and 0.2 N NaOH) for 30 min, and 0.1 mL of the lysate was neutralized with 10 μL 2 N HCl and analyzed by scintillation spectrophotometry in a Tri-Carb B2910TR liquid scintillation analyzer and associated QuantaSmart™ software (version 4.0, PerkinElmer). The remaining lysate was used to measure the cellular protein concentration of each sample by a BCA assay using an Epoch microplate spectrophotometer. Initial rate uptake of glutamine and leucine were calculated from the counts per min (cpm) per sample and the specific activity of each uptake mix (in cpm/nmol), and these measurements were normalized to cellular protein content in a Microsoft Excel spreadsheet. For glutamine uptake studies, transport values obtained in the absence of extracellular Na^+^ (diffusion and Na^+^-independent uptake) were subtracted from those in the presence of Na^+^ (total uptake) to yield Na^+^-dependent rates that are reported in units of nmol·mg^−1^ protein·30 s^−1^. In some experiments, a system A glutamine transport inhibitor, 2-methylaminoisobutyric acid (5 mM, MeAIB; Sigma) and a pan-specific glutamine transport inhibitor, glutaminyl-para-nitroanilide (1 mM, GPNA; MP Biomedicals, Santa Ana, CA, USA) were included in l-[^3^H] Glutamine uptake mixtures to differentiate between system A and system ASC-mediated glutamine transport. Likewise, a LAT1 inhibitor, 2-amino-2-norbornanecarboxlic acid (5 mM, BCH; Sigma) was included in some l-[^3^H] Leucine uptake mixtures to characterize leucine transport mediated by the System L (including LAT1) transporters. All transport values depicted are the average ± SD of four separate determinations. When applicable, to compare transport values between separate cluster plates, non-silencing (NS) controls from each parent cell line were set to a value of 1 and all other transport values were mathematically adjusted to reflect this change.

### 4.7. Glutamine and Leucine Accumulation Analyses

For longer-term accumulation of amino acids, l-[^3^H]Gln or l-[^3^H]Leu were at 3 μCi/mL were added to DMEM (4.5 mg/mL d-glucose) supplemented with 10% triple 0.1 μm filtered dialyzed fetal bovine serum (dFBS) and 100 μM unlabeled l-glutamine. All measurements were carried out at 37 °C and were terminated at specific times (30 s, 1 min, 10 min, 30 min, 1 h, and 1.5 h) by three rapid washes with an ice-cold phosphate-buffered saline (PBS) solution. Free and biosynthetically-incorporated fractions of accumulated radiolabeled amino acid were assessed by acid precipitation. Cells were treated (0.2 mL/well) with 10% (*w/v*) trichloroacetic acid (TCA) (Acros Organics, Morris, NJ, USA) and incubated on ice; after 30 min, 0.1 mL of the supernatant, representing the free (soluble) fraction of radiolabeled amino acid, was removed analyzed by scintillation spectrophotometry. The remaining acid-precipitated cellular macromolecules in each well, representative of the incorporated fraction of radiolabeled amino acid, were washed three times with ice-cold PBS (1 mL/well/wash), solubilized by adding a standard lysis buffer (0.2 mL/well of 0.2% SDS and 0.2 N NaOH), neutralized, and analyzed by scintillation spectrophotometry as previously described. Free radiolabeled amino acid values (nmol AA·mg^−1^ protein) were calculated as the counts per min (cpm) of the supernatant divided by the specific activity of the relevant amino acid uptake mix (glutamine or leucine; cpm/nmol) and normalized to the protein concentration (mg) of each well as determined by a bicinchoninic acid (BCA) assay. Incorporated radiolabeled amino acid values (incorporated nmol AA·mg^−1^ protein) were likewise calculated from the solubilized acid-precipitated fraction. All transport values depicted are the average ± SD of four separate determinations.

### 4.8. Cell Proliferation Assays—MTT Analysis

For methylthiazol tetrazolium (MTT) analysis, SKHep and its derivative cell lines were plated at an initial density of 2.0 × 10^3^ cells/well, and Hep3B, HUH7, and their derivative cell lines were plated at an initial density of 8.0 × 10^3^ cells/well, all in 48-well plates. After cell cultures reached 10–20% confluence (T_0_) before the plates were subjected to MTT analysis at the following time points: T_0_, 24, 48, 72, 96, 120, 144, and 168 h. Cells were washed with PBS and incubated for three hours in a 0.5 mg/mL solution of MTT in phenol red-free DMEM (Gibco) at 37 °C. After incubation, formazan crystals in each well were solubilized with an acidic solution of 0.04 M HCl in absolute isopropanol, and absorbance measured with an Epoch microplate spectrophotometer. Background absorbance was subtracted from each measurement (550–690 nm), and values were calculated as the average ± SD of at least four separate determinations.

### 4.9. mTORC1 Analysis

To evaluate the influence of distinct extracellular signals on mammalian target of rapamycin complex 1 (mTORC1) activity, cells were treated with culture medium starvation and repletion steps, part of a modified protocol based on a procedure described in Nicklin et al., 2009 [16]. Supplemental Figure S16 depicts the order and formulation of each treatment in this analysis. All cell lines were plated at a density of 5.0 × 10^5^ cells/well in 6-well plates, and when this population was 80–90% confluent, the cells were deprived of FBS (serum-free DMEM) for 18–22 h in order to remove growth factor signaling. The cells were next incubated in FBS-free, amino acid-free sodium Krebs-Ringer bicarbonate buffer (NaKRB) (MgSO_4_, KCl, KH_2_PO_4_, NaCl, glucose, NaHCO_3_, phenol red, and CaCl_2_) for 3 h to remove extracellular amino acid signaling. Cells were next differentially treated with: (1) NaKRB + 2 mM l-Gln + essential amino acids (EAA: histidine, isoleucine, leucine, lysine, methionine, phenylalanine, threonine, tryptophan, and valine, all at normal media concentrations); or NaKRB + GLN + EAA plus one of the following four additives: (2) 1 mM GPNA, an ASCT2 inhibitor; (3) 5 mM BCH (Sigma), a LAT1 inhibitor; (4) 20 nM rapamycin (LC Laboratories, Woburn, MA, USA), an mTORC1 inhibitor; or (5) EtOH vehicle control, followed by an incubation time of 1 h at 37 °C. A cellular lysate was prepared from each treatment condition (indicated by the microfuge tube symbol in Supplemental Figure S16) according to the previously described parameters for phosphorylation-specific Western blots in the ‘Western blot Analysis’ section of Materials and Methods.

## Supplementary Materials

Supplementary materials can be found at http://www.mdpi.com/1422-0067/19/7/2093/s1.

## Abbreviations

ASCT2	alanine-serine-cysteine transporter 2
LAT1	large neutral amino acid transporter 1
mTORC1	mammalian or mechanistic target-of-rapamycin (kinase signaling) complex 1
GFP	Green Fluorescence Protein
RT-qPCR	Reverse-Transcription-Quantitative Polymerase Chain Reaction
