# Supplementary material for: Targeted Suppression and Knockout of ASCT2 or LAT1 in Epithelial and Mesenchymal Human Liver Cancer Cells Fail to Inhibit Growth
Source: Int J Mol Sci. 2018 Jul 19;19(7):2093. doi: 10.3390/ijms19072093 (PMC6073291; doi:10.3390/ijms19072093)
Supplement: Supplementary file 1 [file ijms-19-02093-s001.pdf]

SUPPLEMENTAL FIGURES

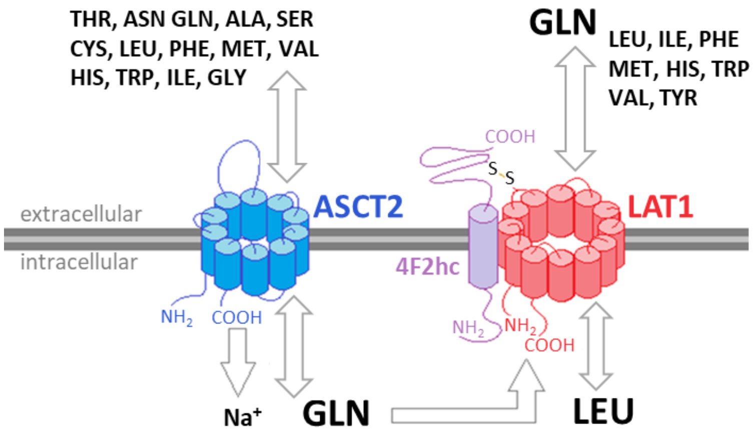

Supplemental Figure 1. ASCT2 and LAT1 transport mechanism. ASCT2 and LAT1mediate a bidirectional transport of amino acids. The coorperative function of these transporters produces an intracellular pool of essential amino acids which stimulates mammalian target-of-rapamycin complex 1 (mTORC1) downstream. *Image created in reference to [1].*

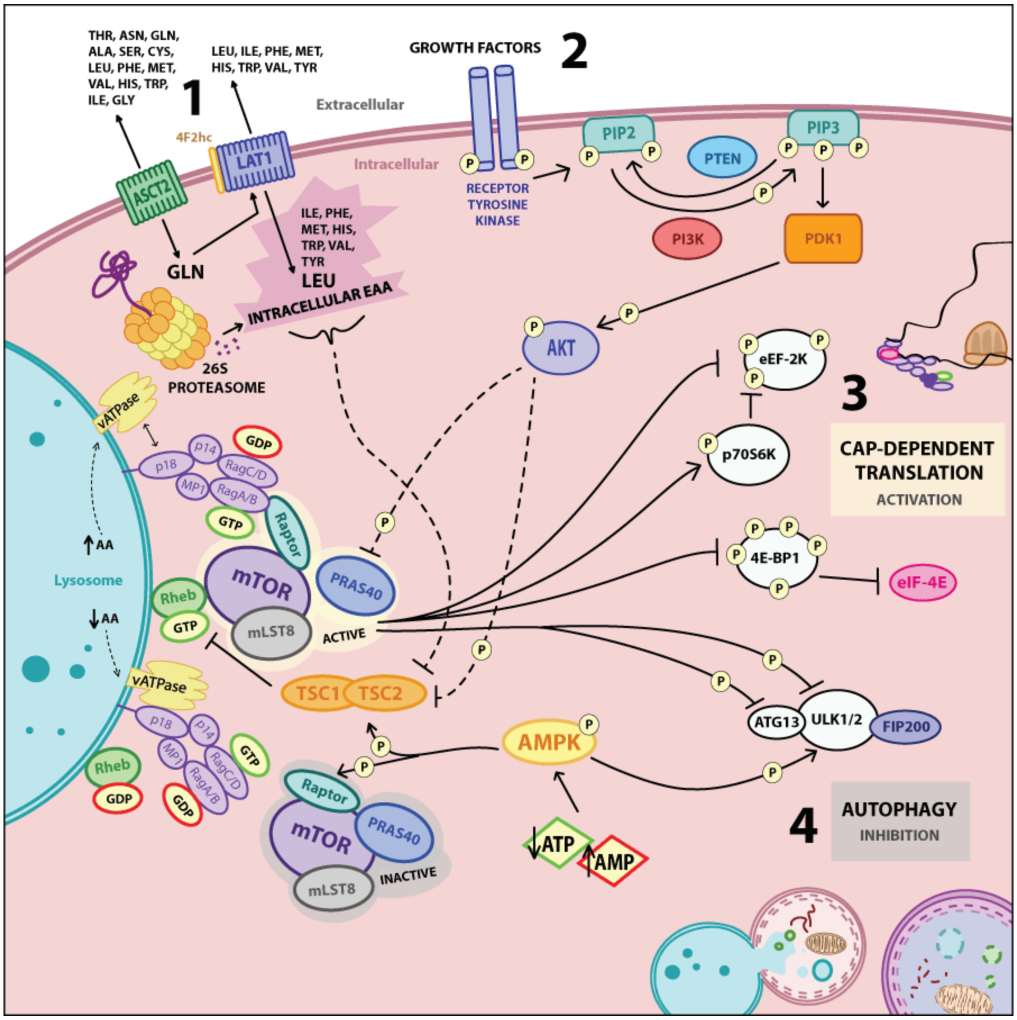

Supplemental Figure 2. Illustration of mammalian target-of-rapamycin complex 1 (mTORC1) activation and activity. Intracellular essential amino acids accrue from the cooperative transport activity of ASCT2 and LAT1 as well as the proteolytic activity of the ubiquitin-proteasome system (**1**) [1-2]. Growth factors bind to receptor tyrosine kinases at the cell surface, and these receptors recruit and activate class I phosphoinositide 3-kinase (PI3K) (**2**). Activated PI3K converts membrane-bound phosphatidylinositol (4,5)-bisphosphate (PIP2) to phosphatidylinositol (3,4,5)-trisphosphate (PIP3), which attracts protein kinase B (PKB/Akt) and phosphoinositide-dependent kinase-1 (PDK1) to the plasma membrane via their pleckstrin homology domains. This recruitment facilitates the phosphorylation and activation of Akt by PDK1 [3]. Upon activation, Akt phosphorylates a broad range of targets including mTORC1 and tuberous sclerosis complex 2 (TSC2) [4-5]. The combined signals from intracellular essential amino acids (**1**) and growth factors (**2**) remove the TSC1/2-mediated inhibition of Ras-homolog-enriched-in-brain (Rheb) [6]. When bound to GTP, Rheb is in its active form and cooperates with the Rag-Ragulator complex to activate and recruit mTORC1 to the lysosomal surface. mTORC1 is a serine threonine kinase that phosphorylates a variety of intracellular targets, including autophagy-related protein 13 (Atg13) and Unc-51 like autophagy activating kinase 1 (ULK1) of the macroautophagic pre-initiation ULK1/2 complex, eukaryotic translation initiation factor 4E-binding protein (4E-BP1), ribosomal protein S6 kinase (p70S6K), and eukaryotic elongation factor-2 kinase (eEF-2K) [7-17]. Together, these phosphorylations lead to the activation of cap-dependent translation (**3**) and the suppression of autophagy (**4**)[18]. In opposition, adenosine monophosphate (AMP)-activated protein kinase (AMPK) also contributes to this dynamic regulation of

intracellular energy homeostasis by phosphorylating raptor on mTORC1, the TSC1/2 complex, and the ULK1/2 complex when the ratio of adenosine triphosphate to adenosine monophosphate (ATP:AMP) is low [19]. This action of AMPK inhibits mTORC1 and activates autophagy. Image created in reference to the respective sources listed above.

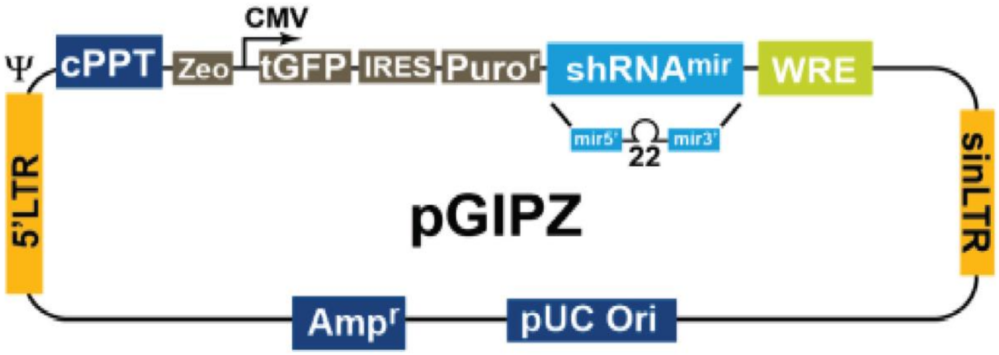

Supplemental Figure 3. Illustration of the GIPZ shRNAmir translentiviral plasmid vector design [20]. Relevant abbreviations: 5′ long terminal repeat (5′LTR), central polypurine tract (cPPT), human cytomegalovirus promoter (CMV), turbo green fluorescent protein (tGFP), internal ribosomal entry site (IRES), puromycin resistance (Puro R), microRNA-adapted shRNA (shRNAmir), woodchuck hepatitis post-transcriptional regulatory element (WRE), 3′ self-inactivating long terminal repeat (sinLTR), amplicillin resistance (Amp R), and bacterial origin of replication (pUC Ori).

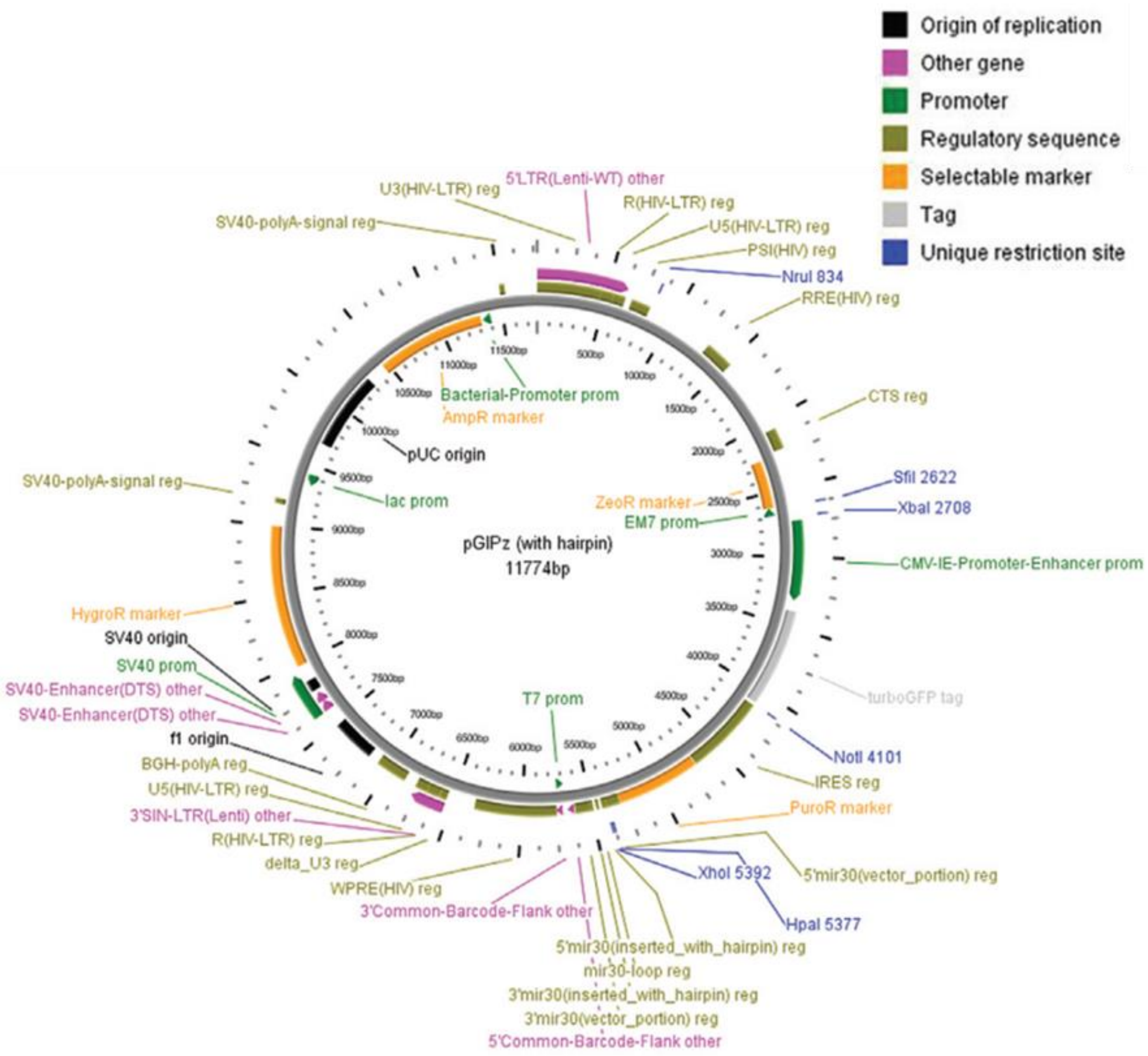

Supplemental Figure 4. Detailed Illustration of the GIPZ shRNAmir translentiviral plasmid vector design [20].

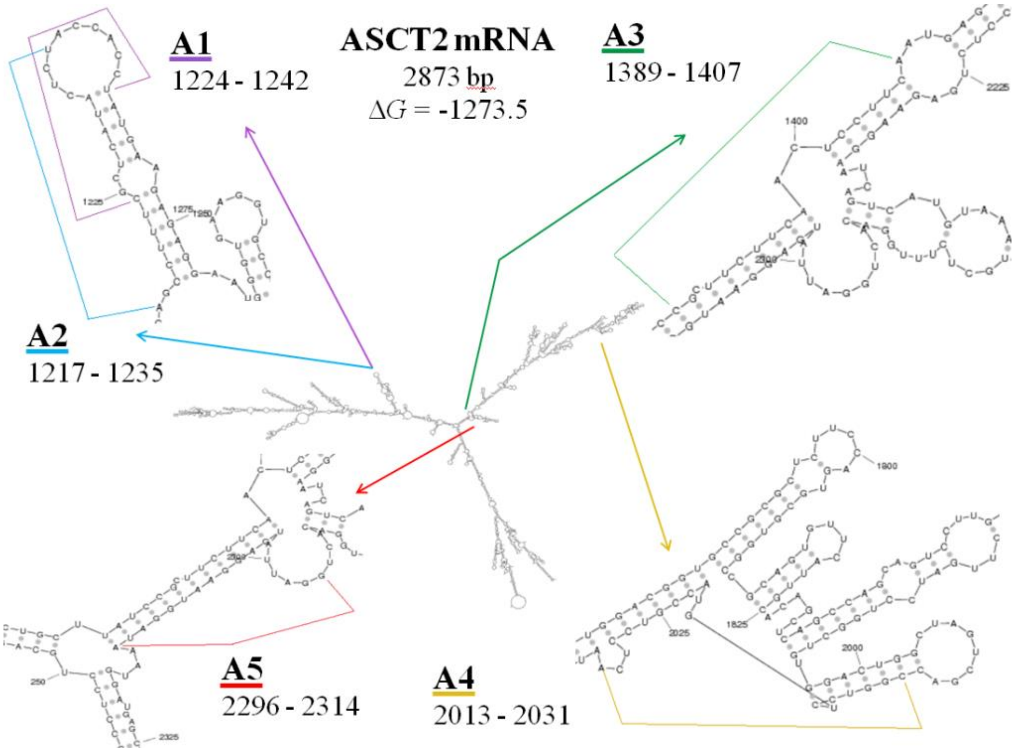

Supplemental Figure 5. shRNAmir target regions on the ASCT2 mRNA. MFE ASCT2 mRNA fold prediction was generated with Sfold software [21-22].

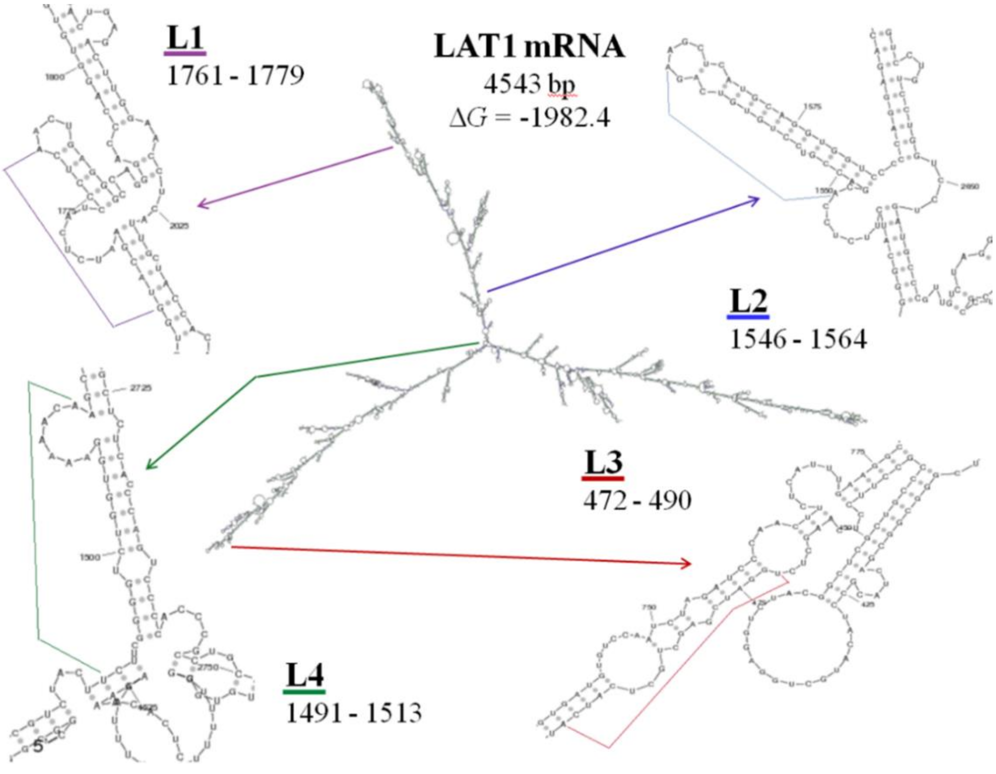

Supplemental Figure 6. shRNAmir target regions on the LAT1 mRNA. MFE LAT1 mRNA fold prediction was generated with Sfold software [21-22].

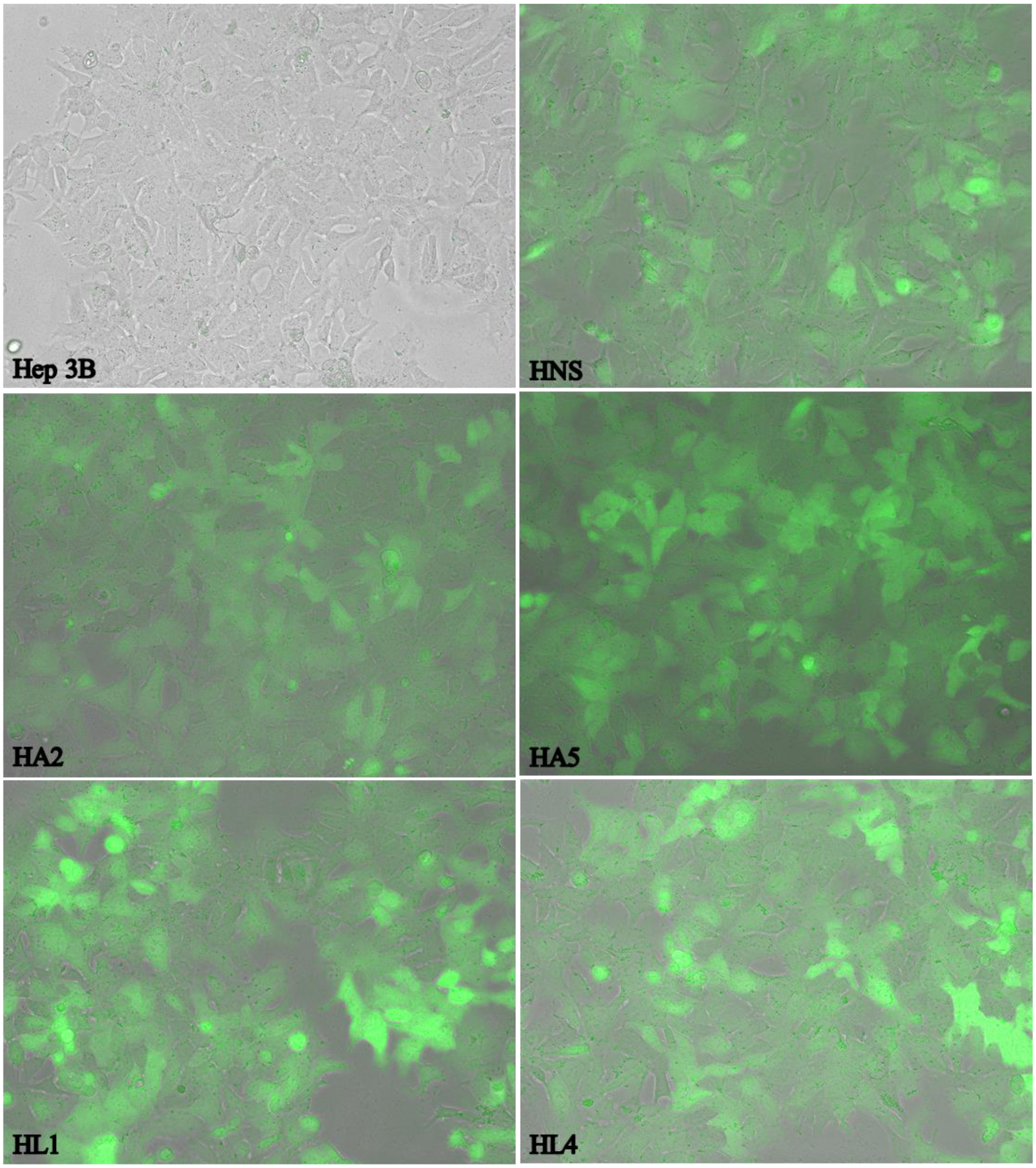

Supplemental Figure 7. tGFP expression in the shRNA-mediated knockdown Hep 3B cell lines. Reporter gene turbo-green fluorescent protein (tGFP; excitation/emission = 482/502 nm) expression in stably transected Hep 3B was visualized using an EVOS® FLoid® cell imaging fluorescent microscope with an 482/18 nm excitation preset. These images are the composite digital overlay of the transmitted light and green fluorescence channels for each cell line.

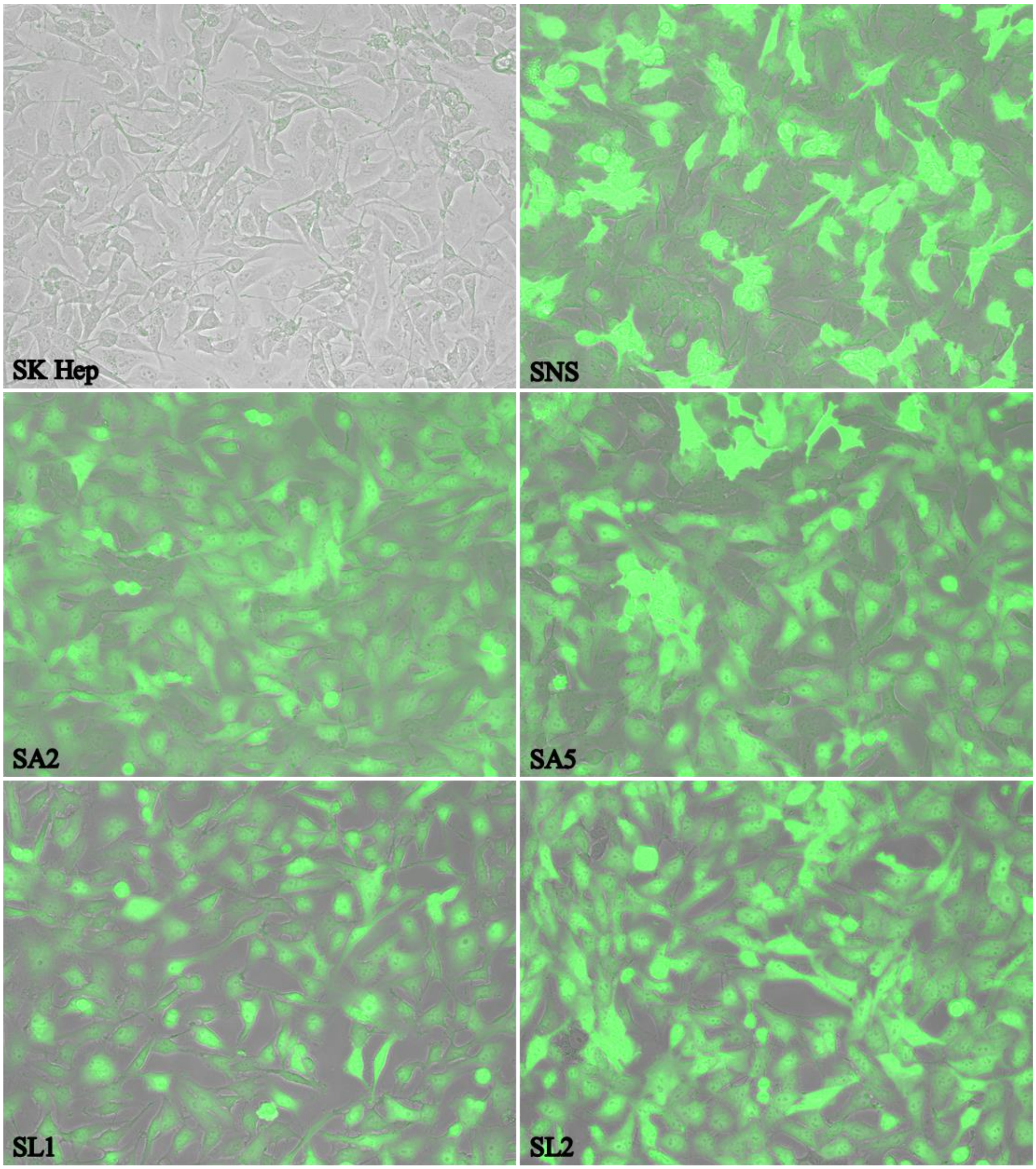

Supplemental Figure 8. tGFP expression in the shRNA-mediated knockdown SK Hep cell lines. Reporter gene turbo-green fluorescent protein (tGFP; excitation/emission = 482/502 nm) expression in stably transected SK Hep was visualized using an EVOS® FLoid® cell imaging fluorescent microscope with an 482/18 nm excitation preset. These images are the composite digital overlay of the transmitted light and green fluorescence channels for each cell line.

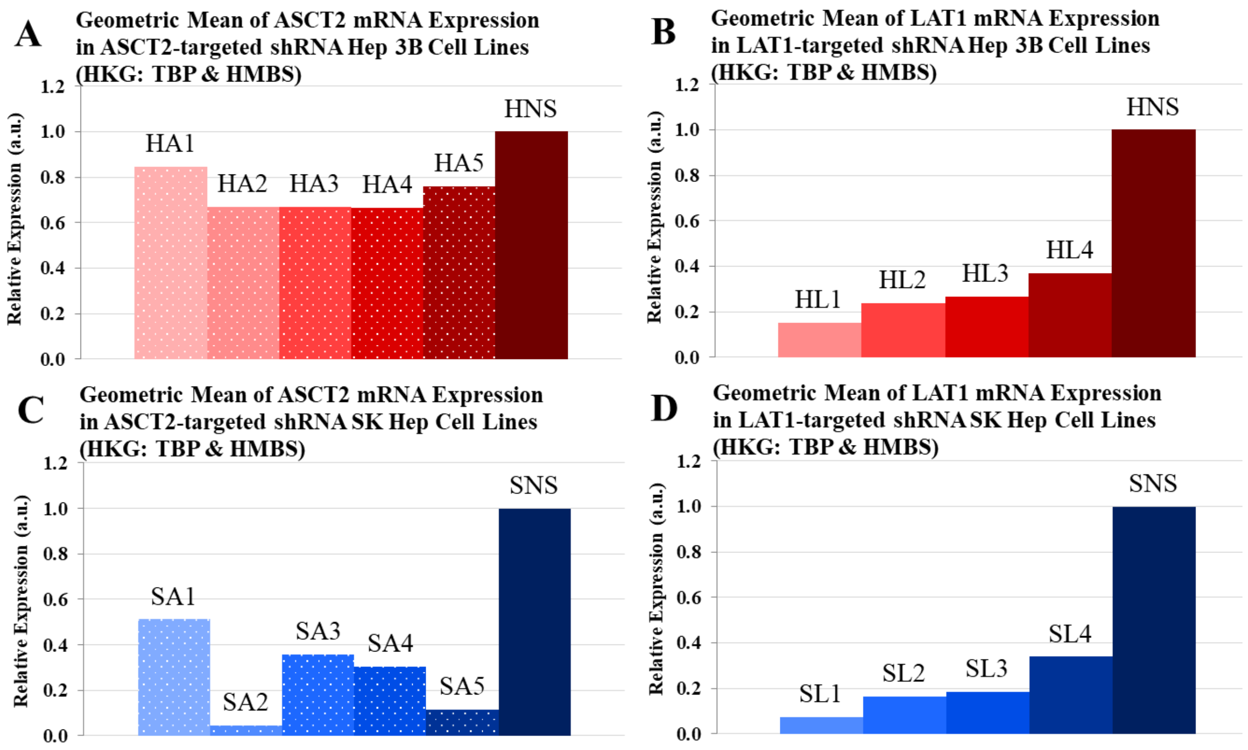

Supplemental Figure 9. ASCT2 and LAT1 mRNA expression after shRNA-mediated knockdown in Hep 3B and SK Hep cell lines. ASCT2 and LAT1 mRNA expression was measured by quantitative RT-PCR analysis as described in Materials and Methods. Each target mRNA expression level is represented as a geometric mean of three experimental RT-qPCR runs relative to the expression level of both TATA-binding protein (TBP) and hydroxymethylbilane synthase (HMBS) housekeeping genes (HKG). (A) Hep 3B-derived ASCT2 knockdown cell lines (HA1-HA5) and (B) LAT1 knockdown cell lines (HL1-HL4) are shown relative to the non-silencing control cell line for Hep 3B (HNS). (C) SK Hep-derived ASCT2 knockdown cell lines (SA1-SA5) and (D) LAT1 knockdown cell lines (SL1-SL4) are shown relative to the non-silencing control cell line for SK Hep (SNS).

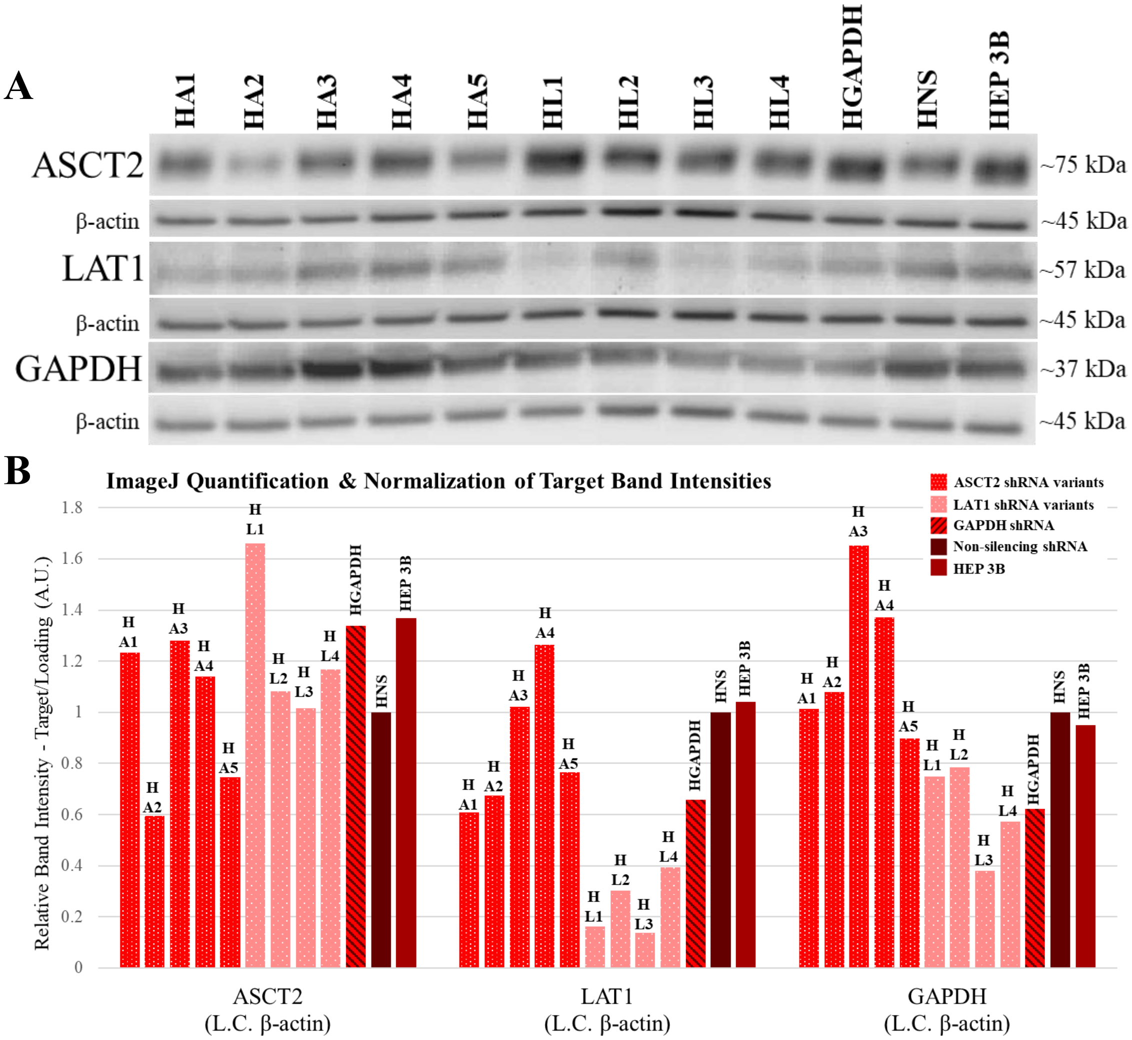

Supplemental figure 10. ASCT2, LAT1, and GAPDH protein expression in shRNA-targeted Hep 3B cell lines. Protein expression was measured by Western blot analysis as described in Materials and Methods. (A) The chemiluminescent bands for each target (ASCT2, LAT1, and GAPDH) are shown in comparison to β-actin protein detected on the same blot in order to control for loading error. (B) The band intensity of each target was measured using ImageJ analysis software and normalized to loading control (L.C.) band intensity in each lane.

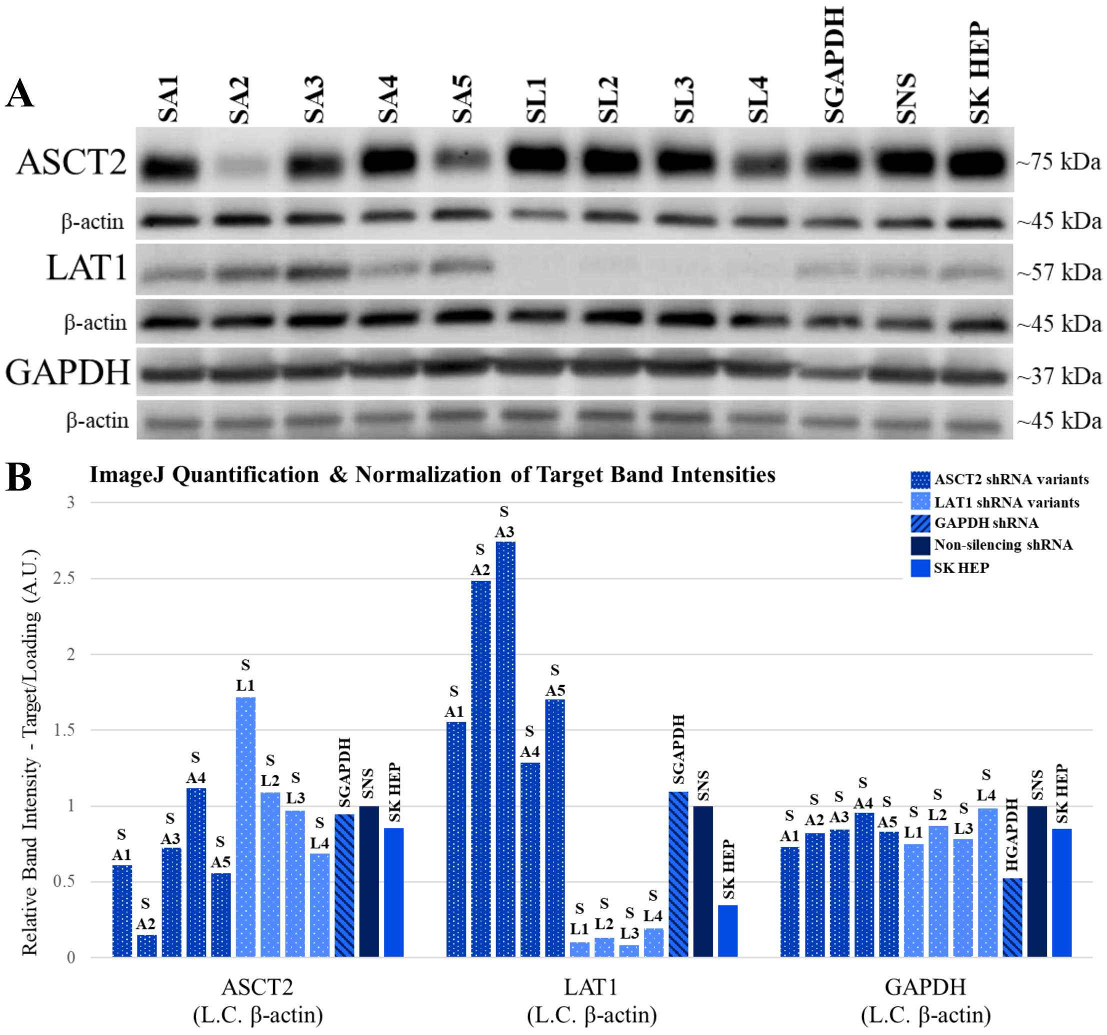

Supplemental Figure 11. ASCT2, LAT1, and GAPDH protein expression in shRNA-targeted SKHep cell lines. Protein expression was measured by Western blot analysis as described in Materials and Methods. (A) The chemiluminescent bands for each target (ASCT2, LAT1, and GAPDH) are shown in comparison to β-actin protein detected on the same blot in order to control for loading error. (B) The band intensity of each target was measured using ImageJ analysis software and normalized to loading control (L.C.) band intensity in each lane.

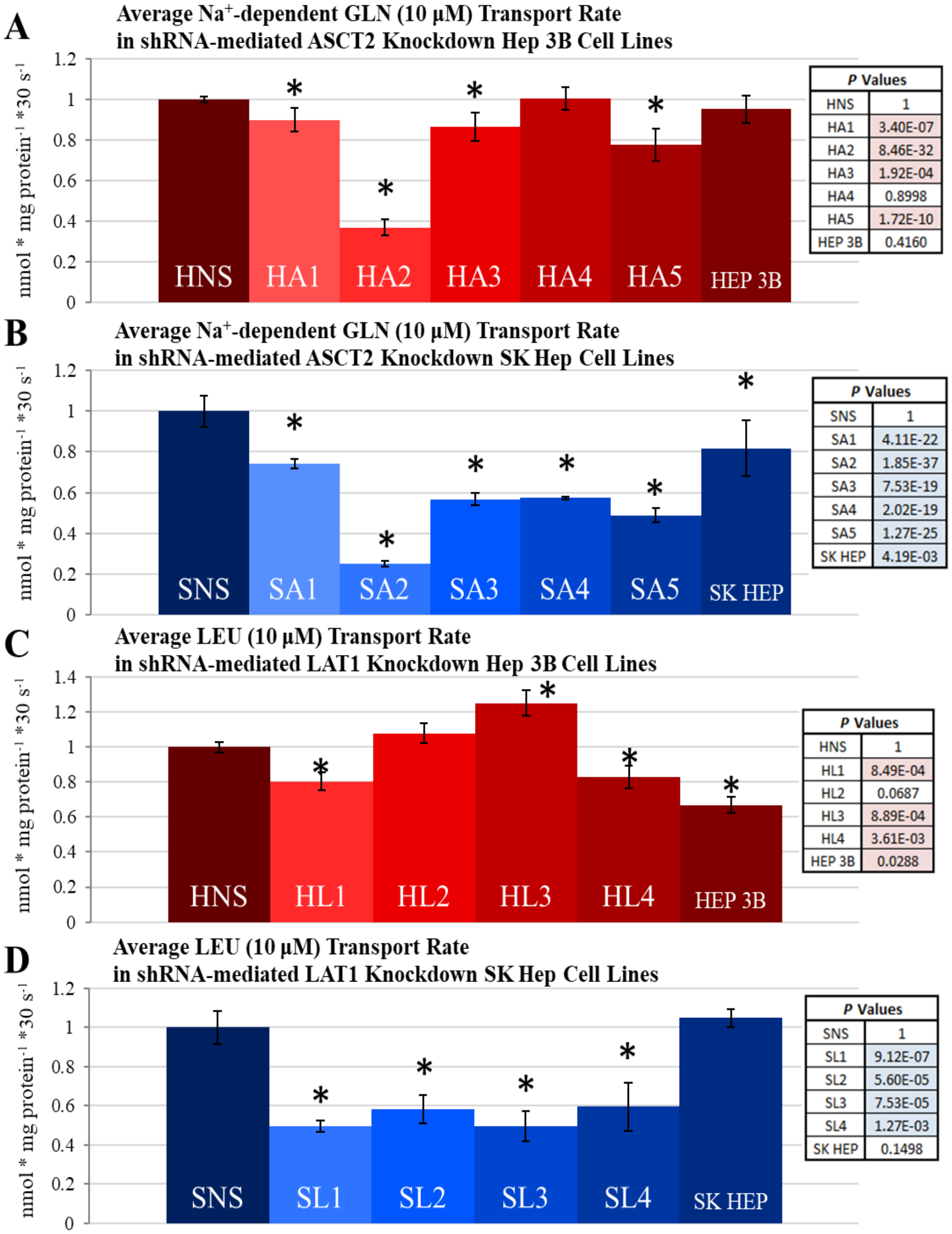

Supplemental Figure 12. Na+-dependent glutamine (L-[3H]Gln) and Na+-independent leucine (L-[3H]Leu) initial rate uptake in shRNA Hep3B and SK Hep cell lines. Transport of 10 µM L-glutamine and 10 µM L-leucine was measured as described in Materials and Methods. Due to the Na+-dependence of ASCT2-mediated glutamine uptake, the transport values obtained in the absence of extracellular Na+ (diffusion and Na+-independent uptake) were subtracted from those in the presence of Na+ (total uptake) to yield Na+-dependent rates depicted in graphs A and B. (A) Hep 3B-derived ASCT2 knockdown cell lines (HA1-HA5) are shown respective to the non-silencing control cell line (HNS) and the Hep 3B parent line; (B) SK Hep-derived ASCT2 knockdown cell lines (SA1-SA5) are shown vs. the non-silencing control cell line (SNS) and the SK Hep parent line. (C) Hep 3B-derived LAT1 knockdown cell lines (HL1-HL4) vs. the non-silencing control cell line (HNS) and the Hep 3B parent line; (D) SK Hep-derived LAT1 knockdown cell lines (SL1-SL4) vs. the non-silencing control cell line (SNS) and the SK Hep parent line. To compare and normalize values between separate cluster plates, NS controls for each parent line were set to a value of 1 and all other transport values were mathematically adjusted to reflect this change. Data are the average of at least four separate determinations ± SD. Asterisks (*) denote values that are statistically significant at $p < 0.050$ vs NS.

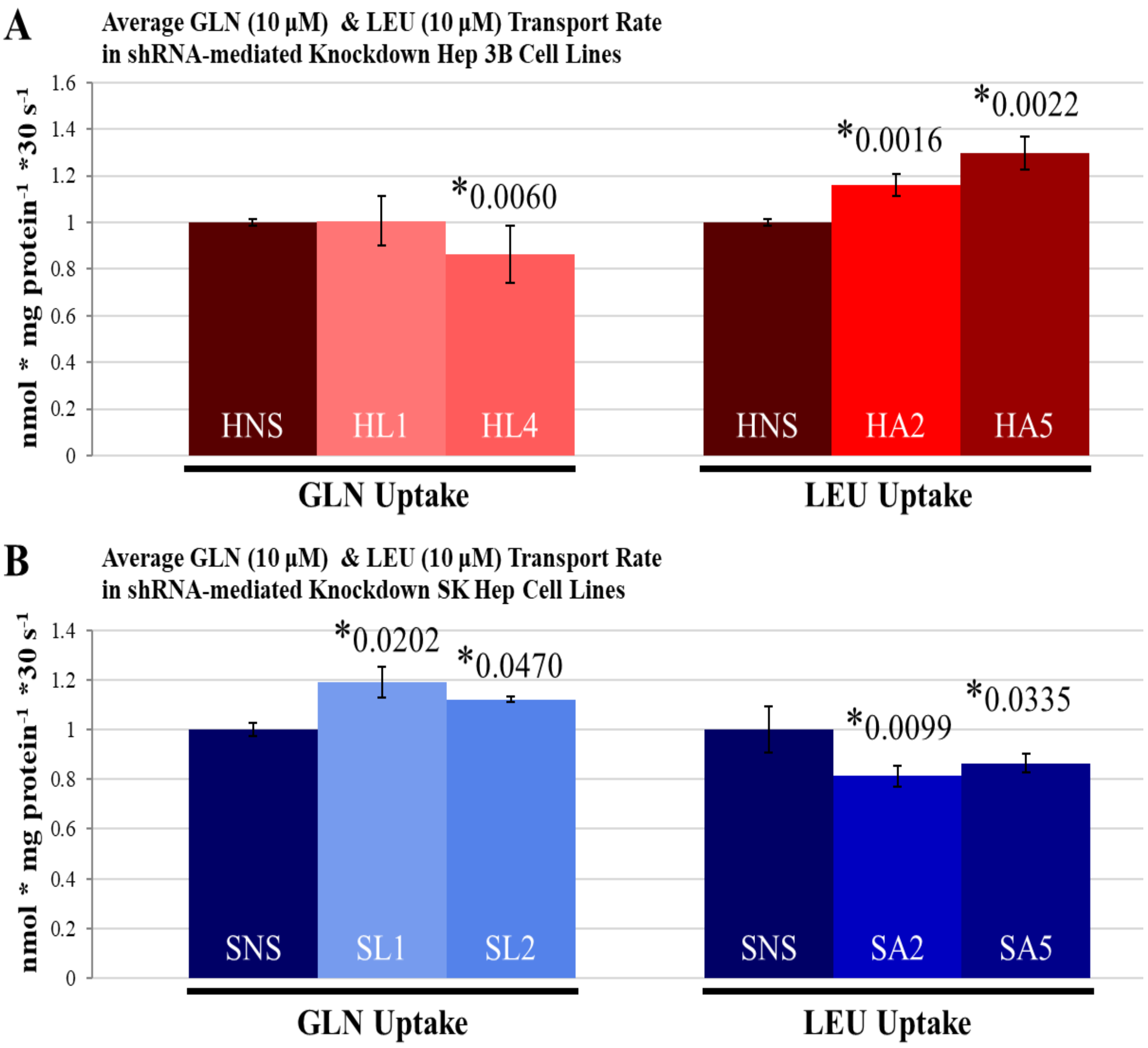

Supplemental Figure 13. Impact of LAT1 suppression on Na+-dependent glutamine (L-[3H]Gln) transport and ASCT2 suppression on leucine (L-[3H]Leu) initial rate uptake. Due to the putative link between of ASCT2 and LAT1 transport activity in the tertiary active transport model, this experiment was performed to determine if suppression of one transporter affects the initial rate uptake activity of the other transporter. (A) Transport of 10 μM L-glutamine and 10 μM L-leucine was measured as described in Materials and Methods. Na+-dependent L-glutamine uptake was measured in two Hep 3B-derived LAT1 knockdown cell lines (HL1 and HL4) and the non-silencing control cell line (HNS), and L-leucine uptake was measured in two Hep 3B-derived ASCT2 knockdown cell lines (HA2 and HA5) and the non-silencing control cell line (HNS). (B) Similarly, Na+-dependent L-glutamine uptake was measured in two SK Hep-derived LAT1 knockdown cell lines (SL1 and SL2) and the non-silencing control cell line (SNS), and L-leucine uptake was also measured in two SK Hep-derived ASCT2 knockdown cell lines (SA2 and SA5) and the non-silencing control cell line (SNS). To compare transport values between separate cluster plates, NS controls for each parent line were set to a value of 1 and all other transport values were mathematically adjusted to reflect this change. Data are the average of at least four separate determinations ± SD. Asterisks (*) denote values that are statistically significant at $p < 0.050$ vs. NS control.

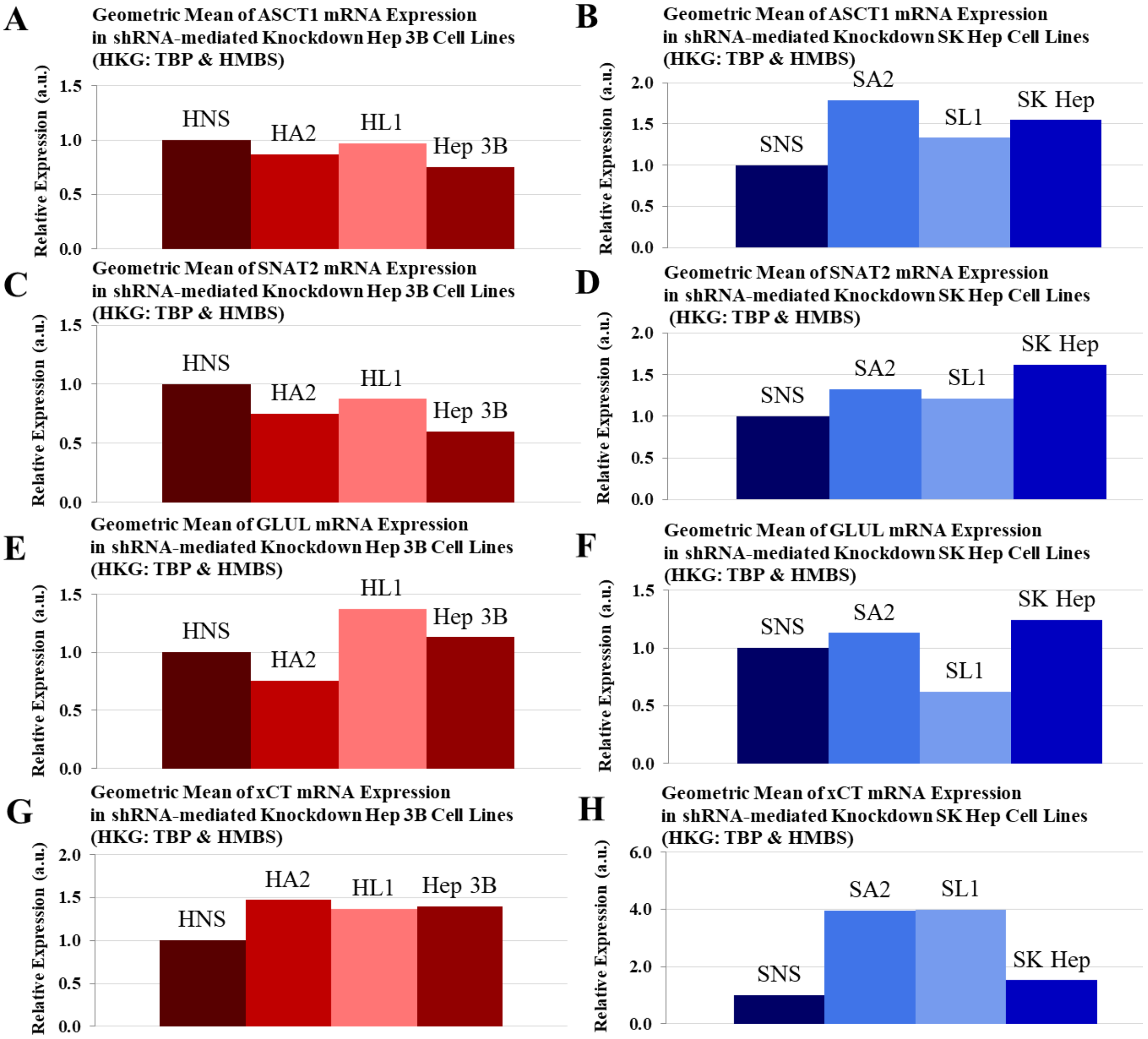

Supplemental Figure 14. ASCT1, SNAT2, GLUL, and xCT mRNA expression in shRNA-mediated transporter suppressed Hep 3B and SK Hep cell lines. mRNA expression was measured by quantitative RT-PCR analysis as described in Materials and Methods. Each target mRNA expression level is represented as a geometric mean of three experimental RT-qPCR runs relative to the expression level of both TBP and HMBS housekeeping genes (HKG). The left-hand column of graphs illustrates target mRNA expression in the shRNA-mediated knockdown Hep 3B cell lines (A, C, E, and G), and the right-hand column of graphs illustrates target mRNA expression in the shRNA-mediated knockdown SK Hep cell lines (B, D, F, and H). (A and B) Expression of ASCT1 (C and D); SNAT2, (E and F); GLUL, (G and H); and xCT mRNA is shown in the ASCT2-targeted cell lines (HA2 and SA2) and LAT1-targeted cell lines (HL1 and SL1) in comparison to their respective non-silencing control cell lines (HNS and SNS) and parent Hep 3B and SKHep cell lines.

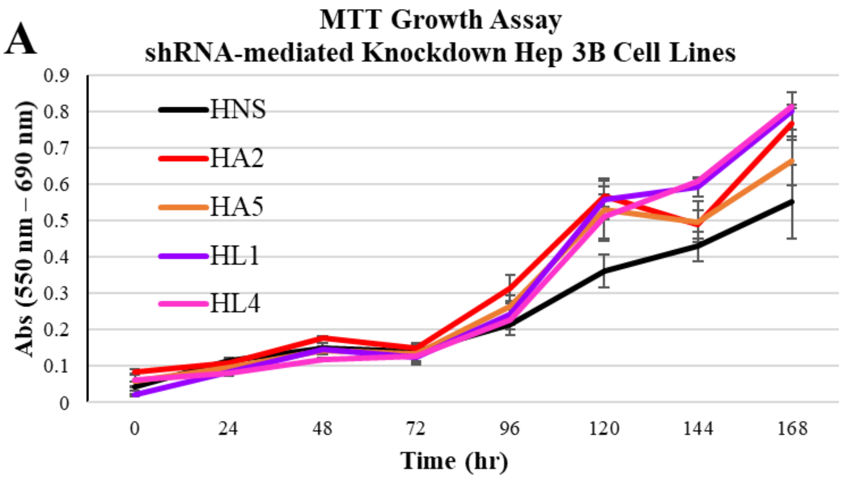

P Values								
	0	24	48	72	96	120	144	168
HNS	1	1	1	1	1	1	1	1
HA2	0.0055	0.4436	0.0161	0.4289	0.0235	0.2099	0.0793	0.0083
HA5	0.3199	0.0053	0.6785	0.7394	0.1230	0.0276	0.1039	0.1172
HL1	0.0140	0.0039	0.9564	0.4311	0.2907	0.0039	0.0005	0.0048
HL4	0.2507	0.0032	0.0306	0.5954	0.7309	0.0263	0.0043	0.0267

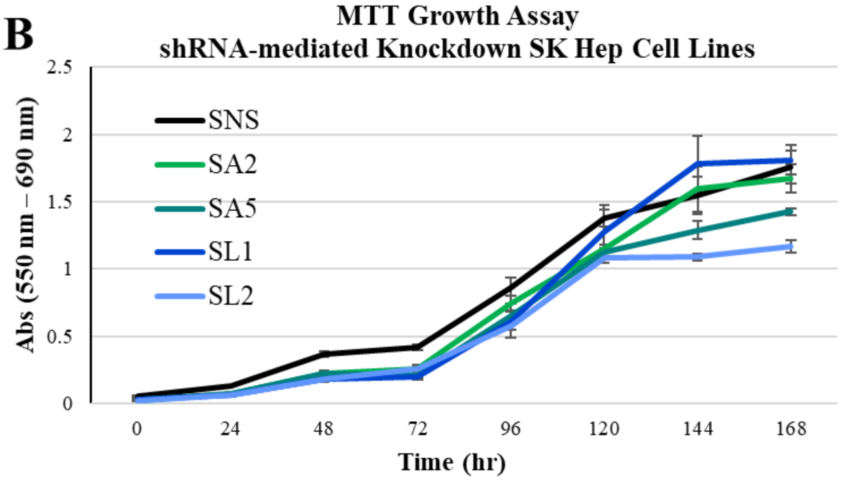

P Values								
	0	24	48	72	96	120	144	168
SNS	1	1	1	1	1	1	1	1
SA2	0.0082	0.0007	0.0001	0.0005	0.0373	0.0007	0.6847	0.3853
SA5	0.4321	0.0000	0.0000	0.0000	0.0013	0.0004	0.0144	0.0108
SL1	0.8305	0.0001	0.0000	0.0000	0.0123	0.3506	0.1053	0.5651
SL2	0.8862	0.0000	0.0000	0.0001	0.0011	0.0002	0.0024	0.0003

Supplemental Figure 15. Growth in the Hep 3B and SK Hep cell lines stably expressing ASCT2 and LAT1 shRNA. Growth was measured using an MTT assay as described in Materials and Methods. (A) Hep 3B-derived ASCT2 and LAT1 knockdown cell lines (HA2, HA5, HL1, and HL4) are shown respective to the non-silencing control cell line (HNS); and the Hep 3B parent line; (B) SK Hep-derived ASCT2 and LAT1 knockdown cell lines (SA2, SA5, SL1, and SL2) are shown respective to the non-silencing control cell line (SNS) and the SKHep parent line.

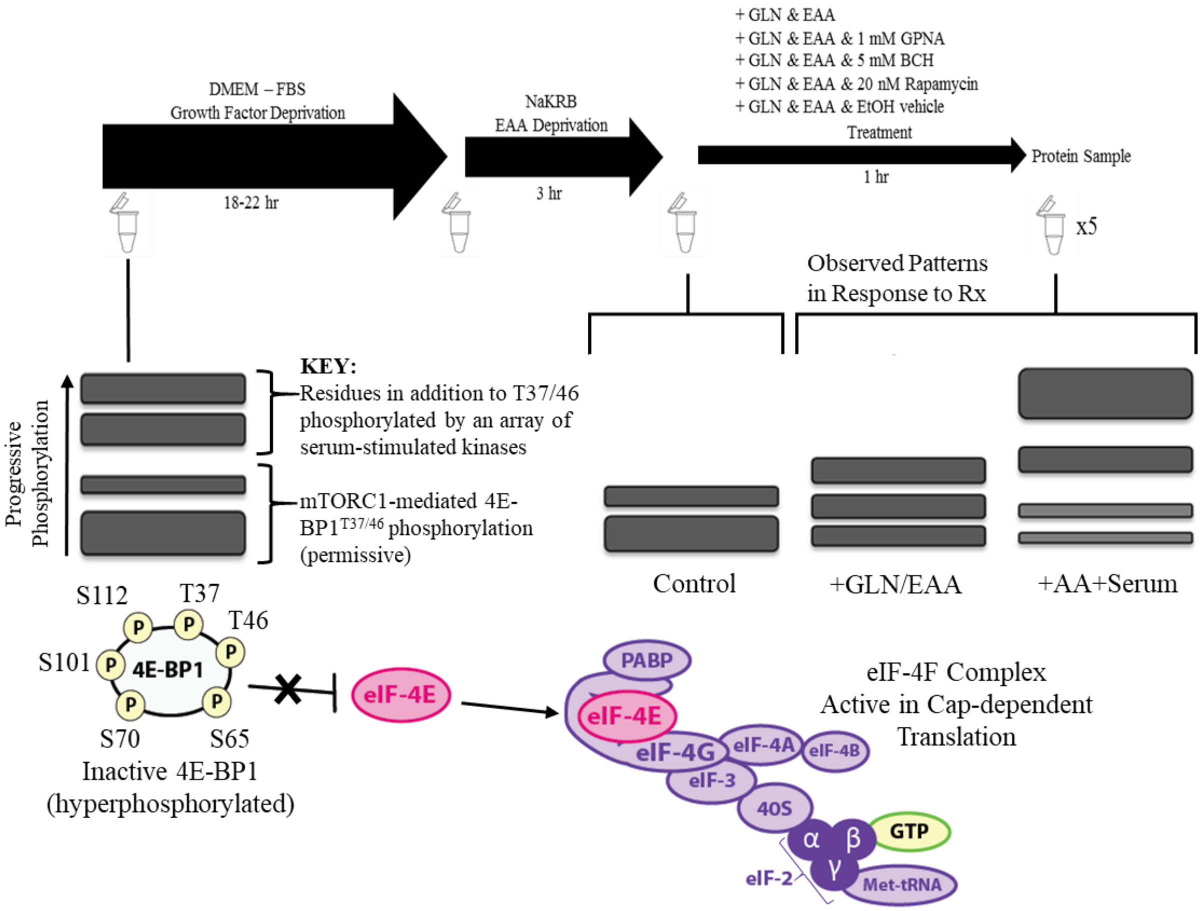

Supplemental Figure 16. mTORC1 treatment steps. Cells were deprived of FBS for 18-22 h to remove extracellular stimulation from growth factors and amino acids, followed by a 3 h amino acid starvation step. Cell lines were then differentially treated with one of five experimental conditions for 1 h. At each stage of the treatment series, total cellular lysate was prepared using a lysis buffer which contained phosphatase inhibitors to preserve the phosphorylation state of all proteins of interest. This protocol is an adaptation of the procedure performed by Nicklin et al., 2009 [23]. The 4E-BP1 phosphorylation-specific Western blots exhibit chemiluminescent bands that have a characteristic stacked appearance, attributable to the fact that 4E-BP1 can exist in a several different phosphorylation states. The top-most band in each 4E-BP1 stack represents the heaviest state, in which 4E-BP1 has the maximum load of phosphorylation, and each band below the top-most band represent consecutively reduced phosphorylation states. Only fully phosphorylated 4E-BP1 is capable of dissociating from eIF-4E, and this dissociation allows eIF-4E to participate in ribosomal recruitment and the assembly of a 5'-cap complex around mRNAs during cap-dependent protein translation [7-9]. The Western blots in this study used an antibody with specificity to 4E-BP1 phosphorylated on threonine 37 and 46 (p-4E-BP1T37/46) mediated directly by mTORC1 kinase activity. These are priming phosphorylations required for subsequent 4E-BP1 phosphorylation by other cellular kinases [14]. Therefore, all bands in the 4E-BP1 stack reactive with p-4E-BP1T37/46 antibody reflect mTORC1 activity, but only the top-most bands in each stack represent a strong signal to cap-dependent translation from the mTORC1 growth pathway. The p-p70S6KT389 antibody implemented in these growth analyses is also specific to a phosphorylation performed by mTORC1 [15]. The phosphorylation on threonine 389 is responsible for priming p70S6K for further phosphorylation by phosphoinositide-dependent kinase-1 (PDK1), ultimately activating p70S6K's kinase activity and subsequent phosphorylation of the S6 ribosomal protein, a step that promotes cap-dependent protein translation. Image created in reference to the respective sources listed above.

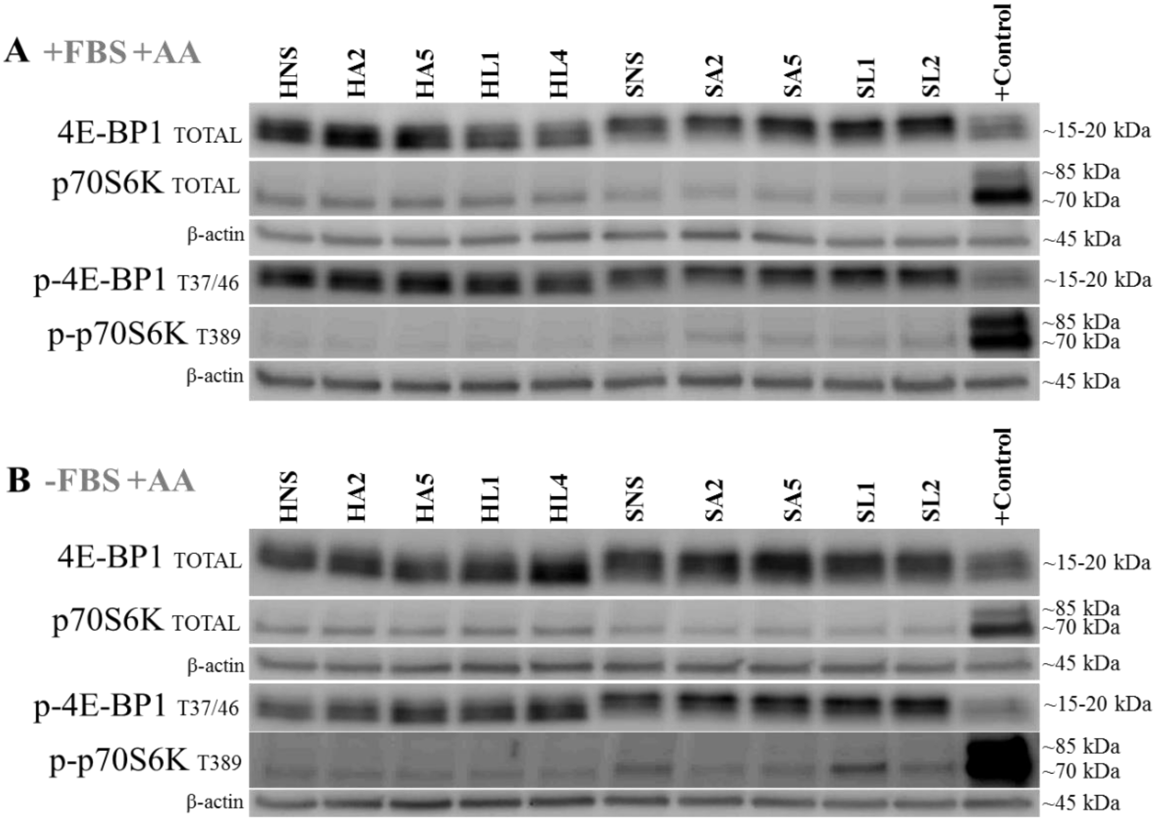

Supplemental Figure 17. The influence of growth factor signaling on 4E-BP1 and p70S6K phosphorylation in the shRNA-suppressed Hep3B and SKHep cell lines. The phosphorylation state of 4E-BP1 and p70S6K was measured by Western blot analysis as described in Materials and Methods. To control for loading, the chemiluminescent bands exposed for each target (total 4E-BP1 and p70S6K; phospho-4E-BP1 and p70S6K) are paired to β-actin protein detected on the same blots, respectively. The last lane of each blot (+Control) is a positive control containing cellular lysate from MCF7 cells treated with insulin. (A) Hep 3B- and SK Hep-derived cell lines maintained under normal growth conditions, exposed to both growth factor signaling via FBS and media levels of amino acids. (B) Hep 3B- and SK Hep-derived cell lines deprived of growth factor signaling (-FBS) for 18-22 h (overnight).

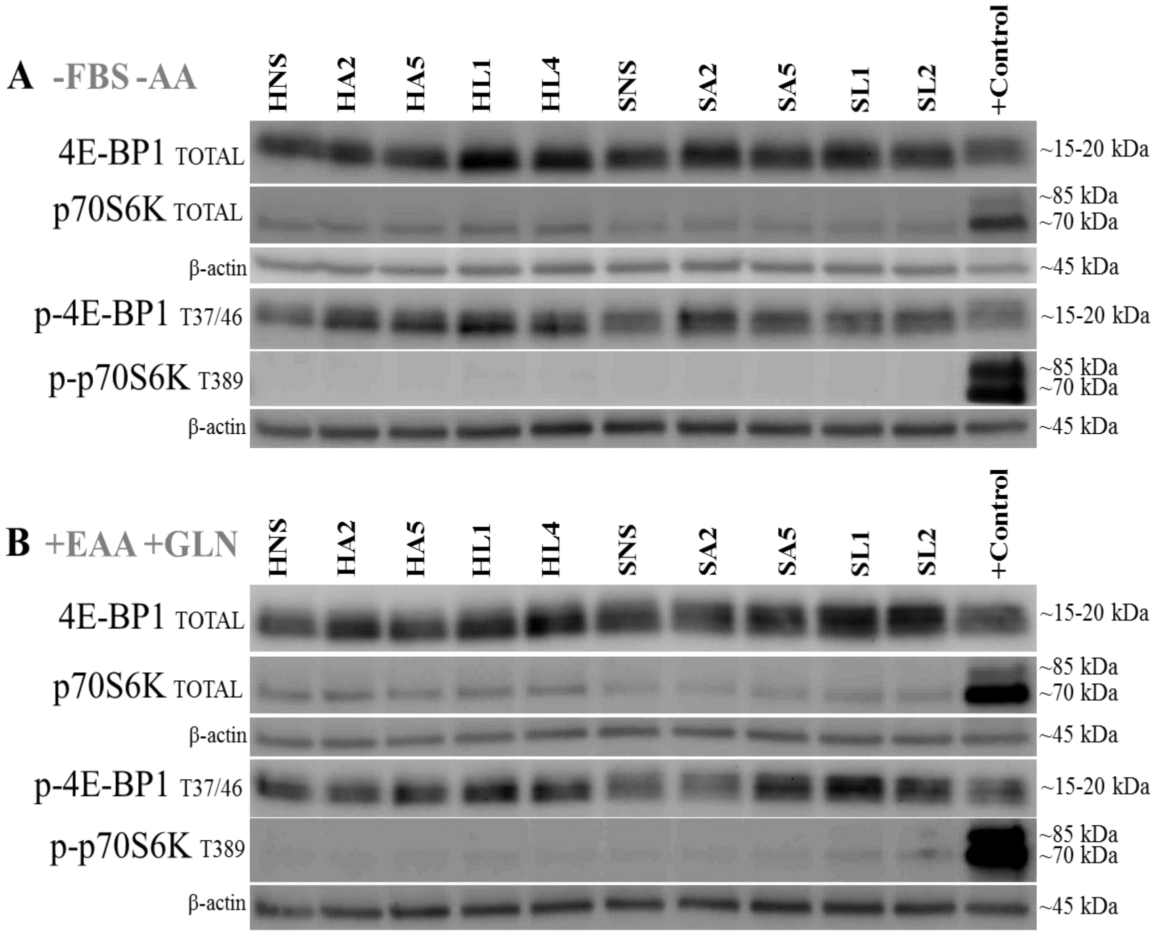

Supplemental Figure 18. Effect of essential amino acids and glutamine on 4E-BP1 and p70S6K phosphorylation in the shRNA-mediated knockdown Hep 3B and SK Hep cell lines, following serum and amino acid deprivation. The phosphorylation state of 4E-BP1 and p70S6K was measured by Western blot analysis as described in Materials and Methods. In order to control for loading, the chemiluminescent bands exposed for each target (total 4E-BP1 and p70S6K; phospho-4E-BP1 and p70S6K) are paired to β-actin protein detected on the same blots, respectively. The last lane of each blot (+Control) is a positive control containing cellular lysate from MCF7 cells treated with insulin. (A) Hep 3B- and SK Hep-derived cell lines deprived of growth factor signaling (-FBS) for 18-22 h (overnight) and then starved for amino acids in a NaKRP buffer for 3 h. (B) Hep 3B- and SK Hep-derived cell lines exposed to the same FBS and amino acid starvation conditions as the first set, but subsequently exposed to essential amino acids and glutamine at normal media concentrations for 1 h.

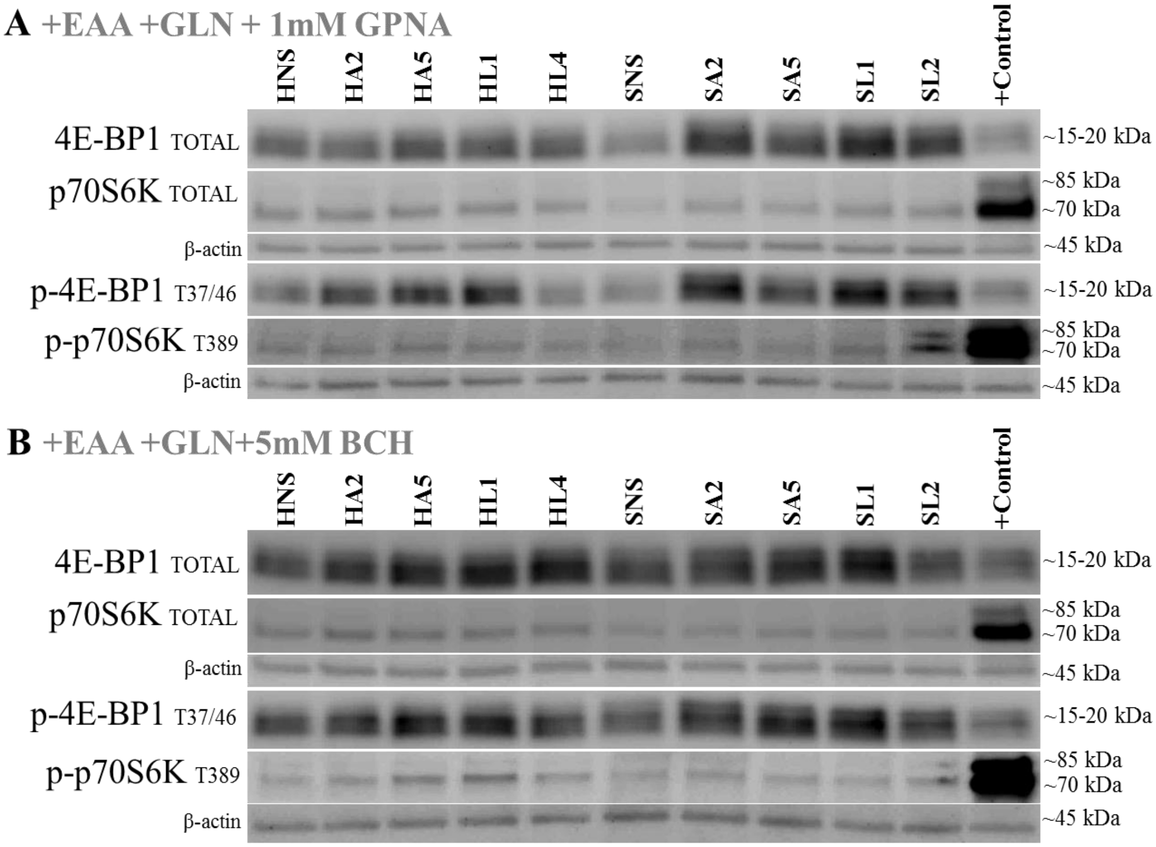

Supplemental Figure 19. Effect of 1 mM GPNA and 5 mM BCH on amino acid-stimulated 4E-BP1 and p70S6K phosphorylation in the shRNA-mediated knockdown Hep 3B and SK Hep cell lines. The phosphorylation state of 4E-BP1 and p70S6K was measured by Western blot analysis as described in Materials and Methods. To control for loading, the chemiluminescent bands exposed for each target (total 4E-BP1 and p70S6K; phospho-4E-BP1 and p70S6K) are paired to β-actin protein detected on the same blots, respectively. The last lane of each blot (+Control) is a positive control containing cellular lysate from MCF7 cells treated with insulin. (A) Hep 3B- and SK Hep-derived cell lines treated with all essential amino acids and glutamine at normal media concentrations plus 1 mM glutaminyl-para-nitroanilide (GPNA), an ASCT2 inhibitor, for 1 h. (B) Hep 3B- and SK Hep-derived cell lines treated with all essential amino acids and glutamine at normal media concentrations plus 5 mM 2-amino-2-norbornanecarboxlic acid (BCH), a system L inhibitor, for 1 h.

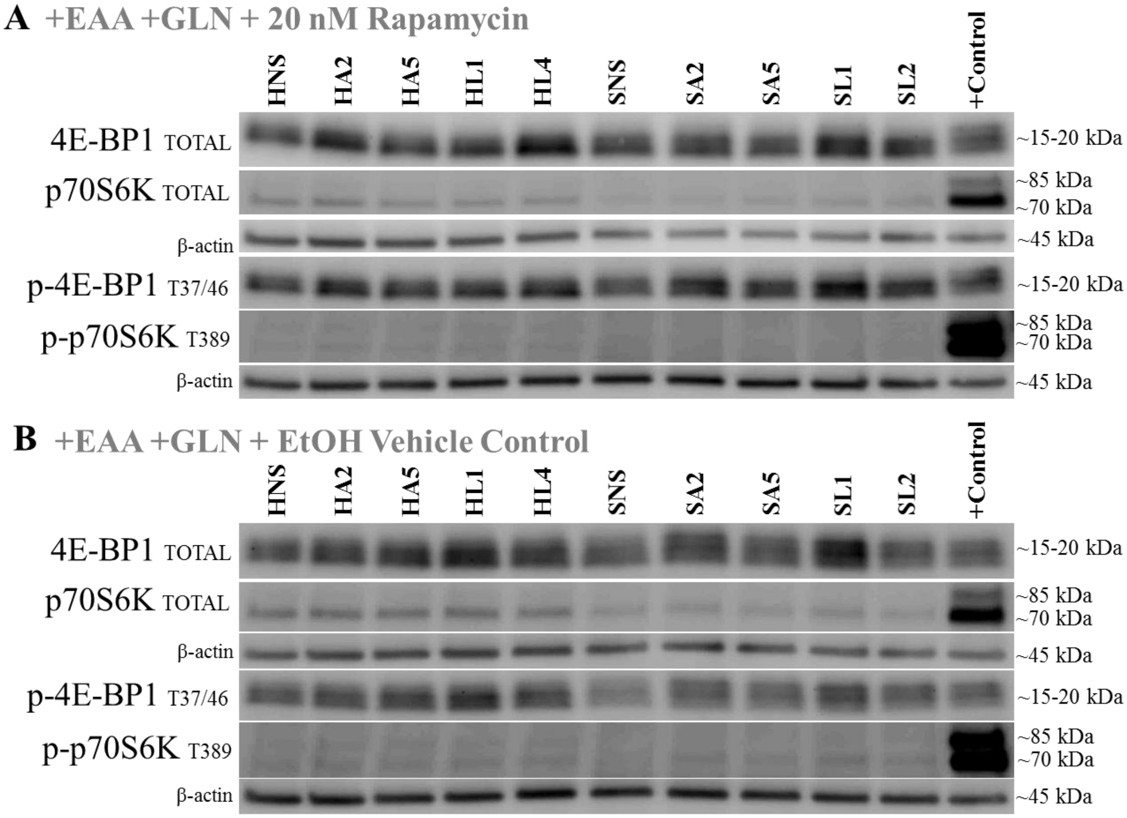

Supplemental Figure 20. Effect of 20 nM rapamycin on amino acid-stimulated 4E-BP1 and p70S6K phosphorylation in the shRNA-mediated knockdown Hep 3B and SK Hep cell lines. The phosphorylation state of 4E-BP1 and p70S6K was measured by Western blot analysis as described in Materials and Methods. To control for loading, the chemiluminescent bands exposed for each target (total 4E-BP1 and p70S6K; phospho-4E-BP1 and p70S6K) are paired to β-actin protein detected on the same blots, respectively. The last lane of each blot (+Control) is a positive control containing cellular lysate from MCF7 cells treated with insulin. (A) Hep 3B- and SK Hep-derived cell lines treated with all essential amino acids and glutamine at normal media concentrations plus 20 nM rapamycin, a mTORC1 inhibitor, for 1 h. (B) Hep 3B- and SK Hep-derived cell lines treated with all essential amino acids and glutamine at normal media concentrations plus an EtOH vehicle control for 1 h.

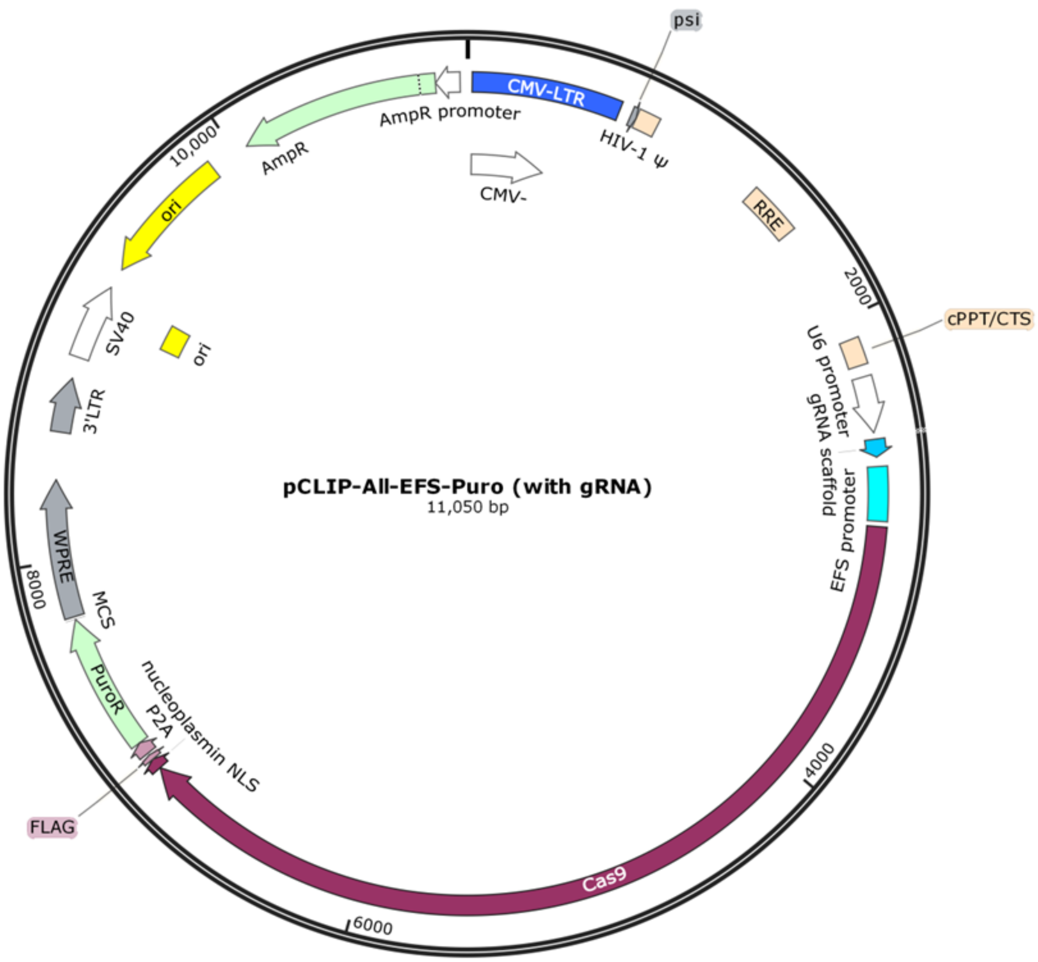

Supplemental Figure 21. pCLIP-All-EFS-Puro CRISPR-Cas9 plasmid vector design [24].

Supplemental Text for section 2.7 CRISPR-Cas9 ASCT2 and LAT1 knockout does not affect mTORC1signaling in epithelial (HUH7) or mesenchymal (SKHep) liver cancer cells. In supplemental Figures 22 and 23, the five vertical lanes for each cell line represent sequential treatment conditions, from left to right: (1) normal growth medium with serum-derived growth factor stimulation (+FBS/+AA), (2) growth medium without serum-derived growth factor stimulation (-FBS/+AA), (3) growth medium without both serum-derived growth factor and amino acid stimulation (-FBS/-AA), (4) normal growth medium with an ethanol (EtOH) vehicle control (+FBS/+AA/+EtOH), and (5) normal growth medium with rapamycin treatment (+FBS/+AA/+20 nM Rapa). Growth signaling analysis was performed as a paired comparison between non-silencing CRISPR-Cas9 control cell lines and the best ASCT2 and LAT1 knockout cell lines derived from HUH7 and SKHep.

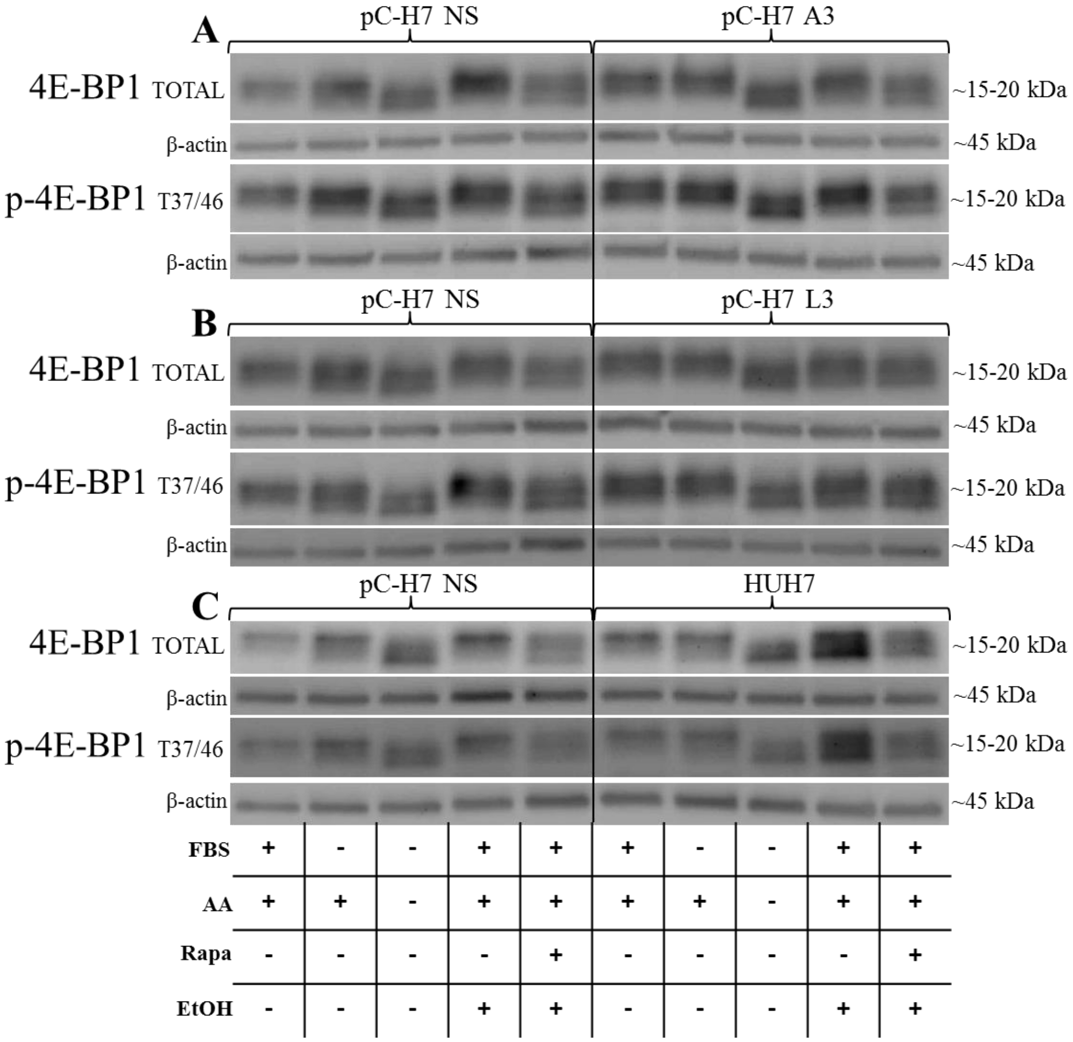

**FBS**	+	-	-	+	+	+	-	-	+	+
**AA**	+	+	-	+	+	+	+	-	+	+
**Rapa**	-	-	-	-	+	-	-	-	-	+
**EtOH**	-	-	-	+	+	-	-	-	+	+

Supplemental Figure 22. The influence of growth factor signaling, amino acid availability, and rapamycin treatment on 4E-BP1 phosphorylation in ASCT2- and LAT1-targeted CRISPR-Cas9 HUH7 cell lines. As a readout of mTORC1 activity, the phosphorylation state of 4E-BP1 was measured by Western blot analysis as described in Materials and Methods. To control for loading, the chemiluminescent bands exposed for each target (total 4E-BP1 and phospho-4E-BP1) are paired to β-actin protein detected on the same blots, respectively. Each array of Western blots is a comparison of 4E-BP1 phosphorylation state changes as these amino acid and serum-deprived cell lines were sequentially exposed for 1 h to the stimulatory and inhibitory conditions described in the table at the bottom of this figure. The non-silencing control cell line (pC-H7 NS) is shown in parallel to (A) ASCT2-targeted HUH7 cell line (pC-H7 A3), (B) the LAT-targeted HUH7 cell line (pC-H7 L3), and (C) the parent HUH7 cell line.

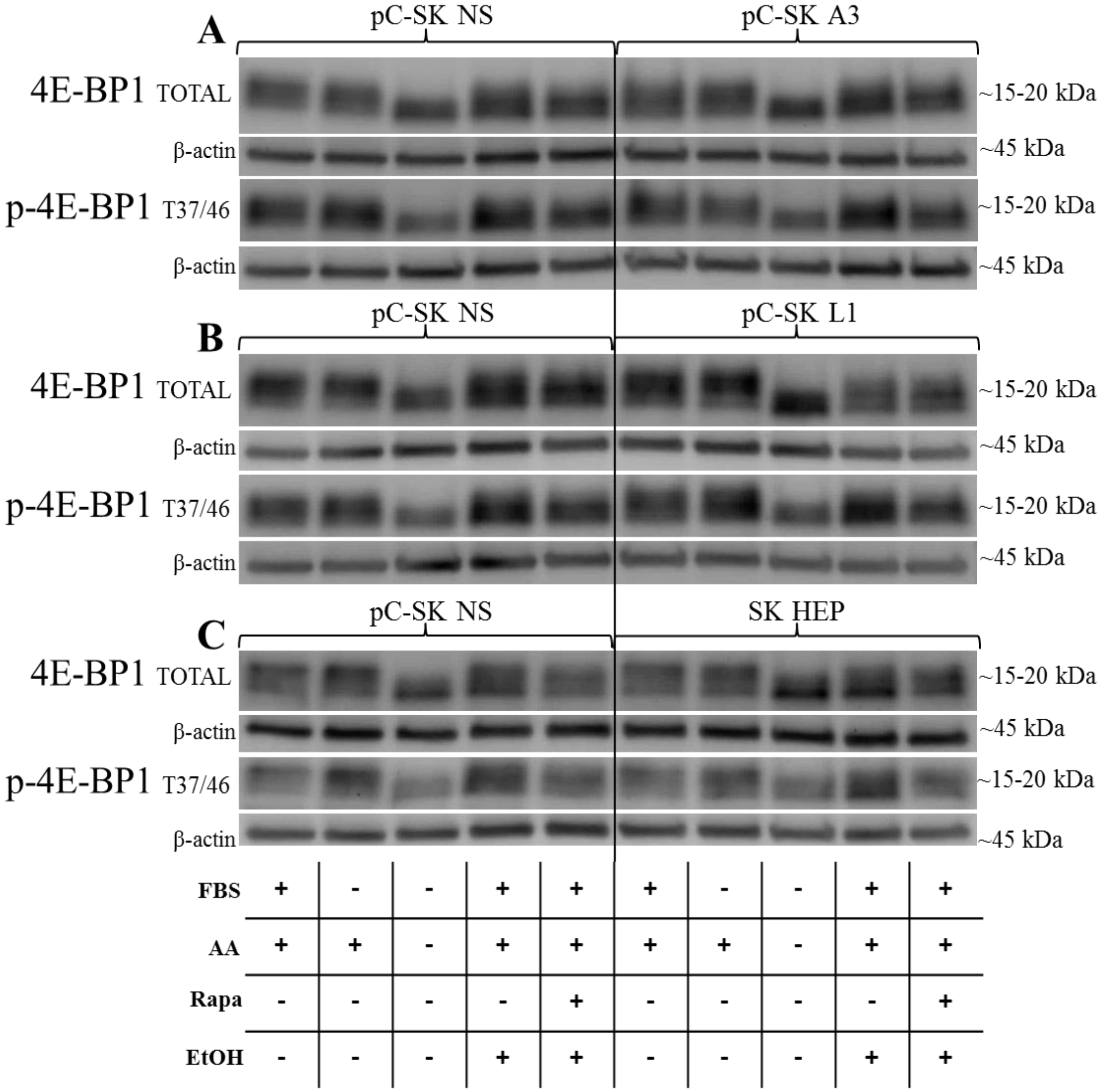

**FBS**	+	-	-	+	+	+	-	-	+	+
**AA**	+	+	-	+	+	+	+	-	+	+
**Rapa**	-	-	-	-	+	-	-	-	-	+
**EtOH**	-	-	-	+	+	-	-	-	+	+

Supplemental Figure 23. The influence of growth factor signaling, amino acid availability, and rapamycin treatment on 4E-BP1 phosphorylation in ASCT2- and LAT1-targeted CRISPR-Cas9 SKHep cell lines. As a readout of mTORC1, the phosphorylation state of 4E-BP1 was measured by Western blot analysis as described in Materials and Methods. In order to control for loading, the chemiluminescent bands exposed for each target (total 4E-BP1 and phospho-4E-BP1) are paired to β-actin protein detected on the same blots, respectively. Each array of Western blots is a comparison of 4E-BP1 phosphorylation state changes as these amino acid and serum-deprived cell lines were sequentially exposed for 1 h to the stimulatory and inhibitory conditions described in the table at the bottom of this figure. The non-silencing control cell line (pC-SK NS) is shown in parallel to the (A) ASCT2-targeted SKHep cell line (pC-SK A3), (**B**) LAT-targeted SKHep cell line (pC-SK L1), and (**C**) the parent SKHep cell line.

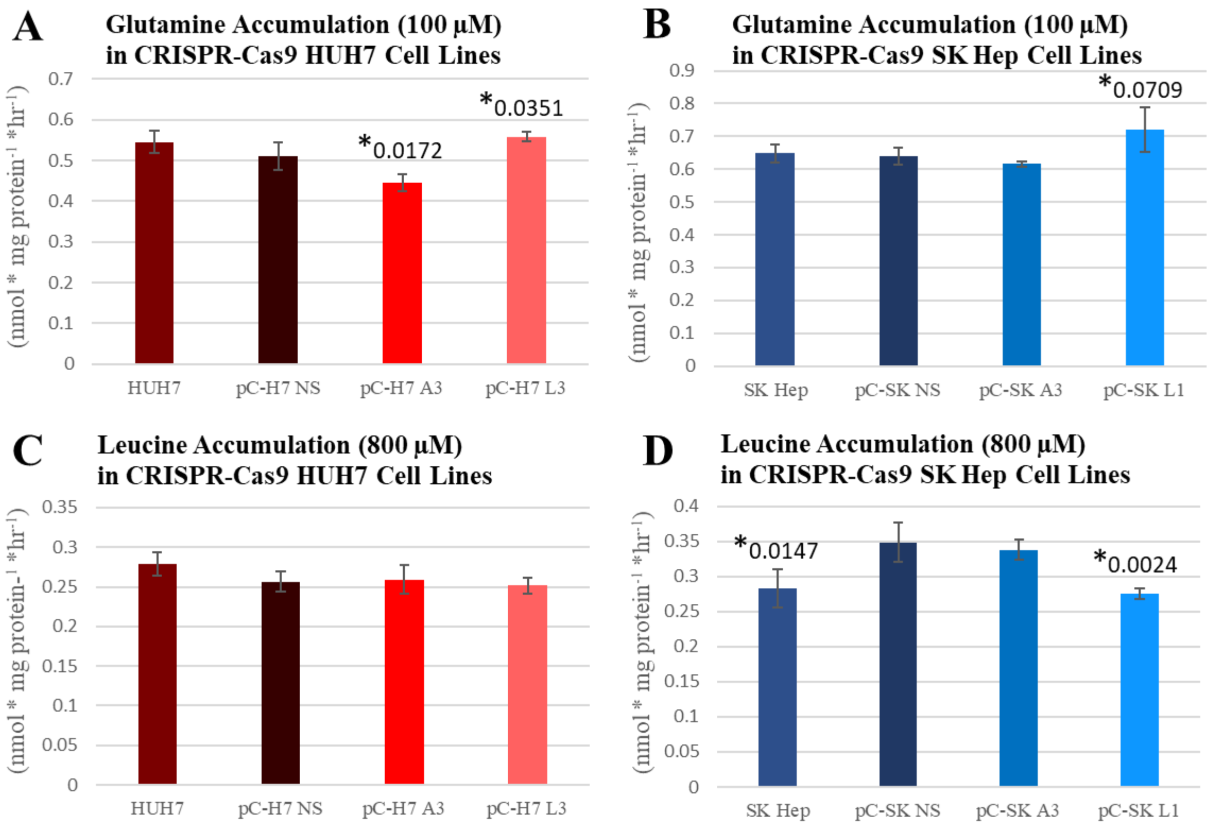

Supplemental Figure 24. Intracellular glutamine (L-[3H]Gln) and leucine (L-[3H]Leu) accumulation over 1 h in ASCT2- and LAT1-targeted CRISPR-Cas9 HUH7 and SK Hep cell lines. Accumulation of 100 µM L-glutamine and 800 µM L-leucine was measured as described in Materials and Methods. (A) Intracellular accumulation of glutamine was measured in ASCT2- and LAT1-targeted HUH7 cell lines (pC-H7 A3 and L3, respectively) compared to the non-silencing control cell line (pC-H7 NS) and parent HUH7cell line, and (B) in ASCT2- and LAT1-targeted SKHep cell lines (pC-SK A3 and L1 respectively, compared to the non-silencing control cell line (pC-SK NS) and parent SKHep cell line. (C) Intracellular accumulation of leucine was measured in ASCT2- and LAT1-targeted HUH7 cell lines (pC-H7 A3 and L3, respectively) compared to the non-silencing control cell line (pC-H7 NS) and parent HUH7cell line, and (D) in ASCT2- and LAT1-targeted SK Hep cell lines (pC-SK A3 and L1 respectively) compared to the non-silencing control cell line (pC-SK NS) and parent SK Hep cell line. Data are the average of at least four separate determinations ± SD. Asterisks (*) denote values that are statistically significant at $p < 0.050$ vs NS control.

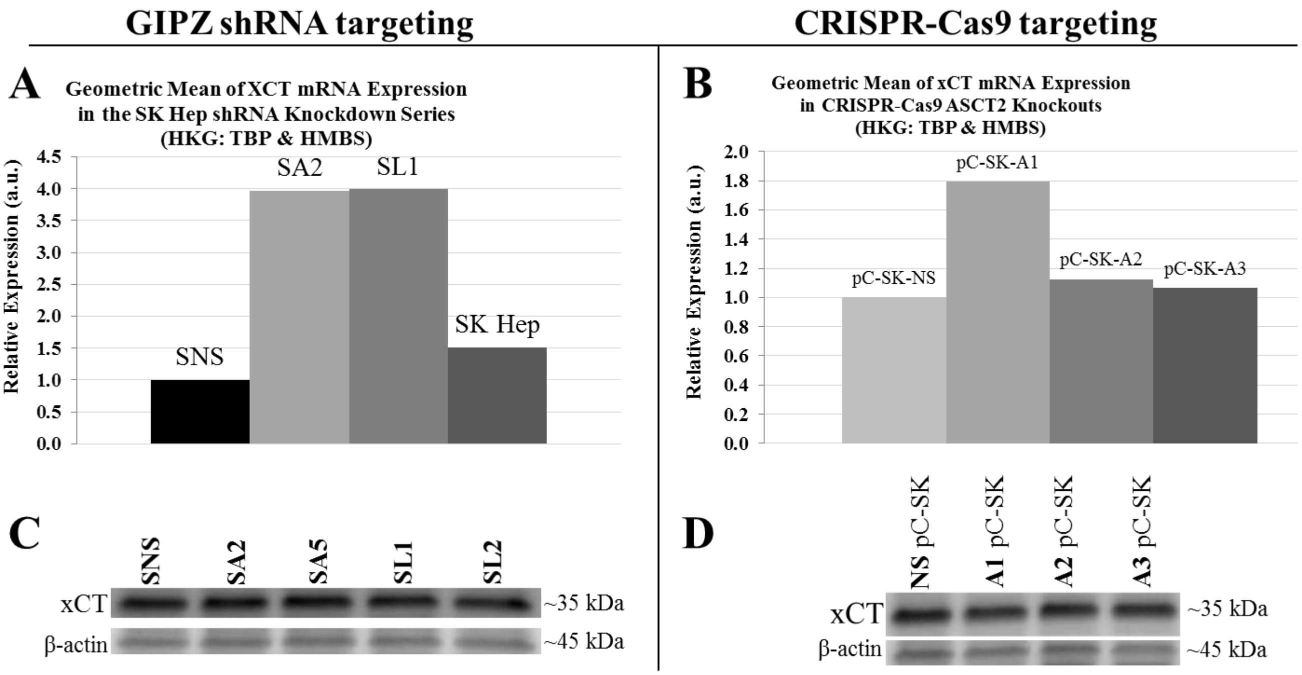

Supplemental Figure 25. xCT mRNA expression in the shRNA-mediated knockdown versus CRISPR-Cas9-mediated knockout SKHep cell lines. (A and B) Cystine-glutamate antiporter (xCT) mRNA expression was measured by quantitative RT-PCR analysis; (C and D) xCT protein expression was measured by Western blot analysis, both as described in Materials and Methods. xCT mRNA expression level is represented as a geometric mean of three experimental RT-qPCR runs relative to the expression level of both TBP and HMBS housekeeping genes (HKG). To control for loading in the Western blot analysis, the chemiluminescent bands exposed for xCT protein are paired to β-actin protein detected on the same blots, respectively. For the shRNA-mediated knockdown cell lines (A and C), the ASCT2- and LAT1-targeted SKHep cell lines (SA2, SA5, SL1 and SL2, respectively) are shown compared to the non-silencing control cell line (SNS) and parent SKHep cell line. (B and D) For the CRISPR-Cas9-mediated knockout cell lines, the ASCT2-targeted SKHep cell lines (pC-SK A1, A2, and A3) are shown compared to the non-silencing control cell line (pC-SK NS).

SUPPLEMENTAL TABLES

Supplemental Table 1. GIPZ shRNAmir sequences and their endogenous mRNA target regions.

**Target Protein** (Gene ID)	**Open Biosystems Clone ID**	**Sense Sequence (5′-3′)** / **Antisense Sequence (5′-3′)**	**mRNA Sequence Targeted**
**ASCT2** (NM_005628)	V3LHS_393625 (A1)	CGCTCATACTCTACCACCT / AGGTGGTAGAGTATGAGCG	1224 – 1242
	V3LHS_393624 (A2)	AGCCTTTCGCTCATACTCT / AGAGTATGAGCGAAAGGCT	1217 – 1235
	V3LHS_393623 (A3)	CGCTTCTTCAACTCCTTCA / TGAAGGAGTTGAAGAAGCG	1389 – 1407
	V3LHS_393622 (A4)	CGGTCCTGTACCGTCCTCA / TGAGGACGGTACAGGACCG	2013 - 2031
	V2LHS_56725 (A5)	GGATTATGAGGAATGGATA / TATCCATTCCTCATAATCC	2296 - 2314
**LAT1** (NM_003486)	V2LHS_172487 (L1)	GTACGAATCTCATCCCTCA / TGAGGGATGAGATTCGTAC	1761 - 1779
	V3LHS_353675 (L2)	ACGACCGTCCTGTGTCAGA / TCTGACACAGGACGGTCGT	1546 - 1564
	V3LHS_353676 (L3)	TGGATCGAGCTGCTCATCA / TGATGAGCAGCTCGATCCA	472 - 490
	V3LHS_353679 (L4)	GGGGTCTGGTGGAAAAACA / TGTTTTTCCACCAGACCCC	1491 - 1513
**GAPDH** (NM_002046.3)	V3LHS_382663 (GAPDH)	TGGTTTACATGTTCCAATAT / ATATTGGAACATGTAAACCA	230-248
**Non-Silencing**	CAT#:RHS4346 (NS)	ATCTCGCTTGGGCGAGAGTAAG / CTTACTCTCGCCCAAGCGAGAG	N/A

Supplemental Table 2. CRSIPR-Cas9 gRNA sequences and their endogenous gene target regions.

**Target Gene Symbol**	**CRISPR Clone ID**	**gRNA sequence (no PAM) (5′-3′)** *SLC1A5* (ASCT2) NC_000019.10 Chrom.19: 46,774,883-46,788,594 *SLC7A5* (LAT1) NC_000016.10 Chrom.16: 87,830,023-87,869,488	**Target Type**
SLC1A5	TEVH-1126600 (A1)	**TGGCAAACACTACCAAGCCC** [46,794,787-46,794,807]	Antisense
SLC1A5	TEVH-1193742 (A2)	**GGTCTCCTGGATCATGTGGT** [46,794,898-46,794,918]	Sense, intron-exon boundary
SLC1A5	TEVH-1260884 (A3)	**CCACGATCTTGCCAGCCACC** [46,798,417-46,798,437]	Antisense
SLC7A5	TEVH-1112259 (L1)	**GATGCTGGCCGCCAAGAGCG** [87,869,646-87,869,666]	Sense
SLC7A5	TEVH-1179401 (L2)	**ATTGTGCTGGCATTATACAG** [87,897,900-87,897,920]	Sense
SLC7A5	TEVH-1246543 (L3)	**CGGAACATCACGCTGCTCAA** [87,869,713-87,869,733]	Sense
TELA1011	Non-silencing	**GGAGCGCACCATCTTCTTCA**	N/A

SUPPLEMENTAL REFERENCES

1. Fuchs, B.C.; Bode, B.P. Amino acid transporters ASCT2 and LAT1 in cancer: Partners in crime? *Semin. Cancer Biol.* **2005**, *15*, 254–266.
2. Wu, W.K.; Volta, V.; Cho, C.H.; Wu, Y.C.; Li, H.T.; Yu, L.; Li, Z.J.; Sung, J.J. Repression of protein translation and mTOR signaling by proteasome inhibitor in colon cancer cells. *Biochem. Biophys. Res. Commun.* **2009**, *386*, 598–601.
3. Wullschleger, S.; Loewith, R.; Hall, M.N. TOR signaling in growth and metabolism. *Cell* **2006**, *124*, 471–484.
4. Inoki, K.; Li, Y.; Zhu, T.; Wu, J.; Guan, K.L. TSC2 is phosphorylated and inhibited by Akt and suppresses mTOR signalling. *Nat. Cell Biol.* **2002**, *4*, 648–657.
5. Nascimento, E.B.; Snel, M.; Guigas, B.; van der Zon, G.C.; Kriek, J.; Maassen, J.A.; Jazet, I.M.; Diamant, M.; Ouwens, D.M. Phosphorylation of PRAS40 on Thr246 by PKB/AKT facilitates efficient phosphorylation of Ser183 by mTORC1. *Cell. Signal.* **2010**, *22*, 961–967.
6. Inoki, K.; Zhu, T.; Guan, K.L. TSC2 mediates cellular energy response to control cell growth and survival. *Cell* **2003**, *115*, 577–590.
7. Brown, E.J.; Beal, P.A.; Keith, C.T.; Chen, J.; Shin, T.B.; Schreiber, S.L. Control of p70 s6 kinase by kinase activity of FRAP in vivo. *Nature* **1995**, *377* (6548), 441-446.
8. Brunn, G.J.; Hudson, C.C.; Sekulic, A.; Williams, J.M.; Hosoi, H.; Houghton, P.J.; Lawrence, J.C., Jr.; Abraham, R.T., Phosphorylation of the translational repressor PHAS-I by the mammalian target of rapamycin. *Science* **1997**, *277*, 99–101.
9. Hosokawa, N.; Hara, T.; Kaizuka, T.; Kishi, C.; Takamura, A.; Miura, Y.; Iemura, S.; Natsume, T.; Takehana, K.; Yamada, N.; et al. Nutrient-dependent mTORC1 association with the ULK1-Atg13-FIP200 complex required for autophagy. *Mol. Biol. Cell* **2009**, *20*, 1981–1991.
10. Jung, C.H.; Jun, C.B.; Ro, S.H.; Kim, Y.M.; Otto, N.M.; Cao, J.; Kundu, M.; Kim, D.H. ULK-Atg13-FIP200 complexes mediate mTOR signaling to the autophagy machinery. *Mol. Biol. Cell* **2009**, *20*, 1992–2003.
11. Kim, J.; Kundu, M.; Viollet, B.; Guan, K.L. AMPK and mTOR regulate autophagy through direct phosphorylation of Ulk1. *Nat. Cell Biol.* **2011**, *13*, 132–141.
12. Svitkin, Y.V.; Herdy, B.; Costa-Mattioli, M.; Gingras, A.C.; Raught, B.; Sonenberg, N. Eukaryotic translation initiation factor 4E availability controls the switch between cap-dependent and internal ribosomal entry site-mediated translation. *Mol. Cell. Biol.* **2005**, *25*, 10556–10565.
13. Martineau, Y.; Azar, R.; Bousquet, C.; Pyronnet, S. Anti-oncogenic potential of the eIF4E-binding proteins. *Oncogene* **2013**, *32*, 671–677.
14. Gingras, A.C.; Gygi, S.P.; Raught, B.; Polakiewicz, R.D.; Abraham, R.T.; Hoekstra, M.F.; Aebersold, R.; Sonenberg, N. Regulation of 4E-BP1 phosphorylation: A novel two-step mechanism. *Genes Dev.* **1999**, *13*, 1422–1437.
15. Pullen, N.; Thomas, G. The modular phosphorylation and activation of p70s6k. *FEBS Lett.* **1997**, *410*, 78–82.
16. Redpath, N.T.; Foulstone, E.J.; Proud, C.G. Regulation of translation elongation factor-2 by insulin via a rapamycin-sensitive signalling pathway. *EMBO J.* **1996**, *15*, 2291–2297.
17. Wang, L.; Harris, T.E.; Roth, R.A.; Lawrence, J.C., Jr. PRAS40 regulates mTORC1 kinase activity by functioning as a direct inhibitor of substrate binding. *J. Biol. Chem.* **2007**, *282*, 20036–20044.
18. Silvera, D.; Formenti, S.C.; Schneider, R.J. Translational control in cancer. *Nat. Rev. Cancer* **2010**, *10*, 254–266.
19. Mihaylova, M.M.; Shaw, R.J. The AMPK signalling pathway coordinates cell growth, autophagy and metabolism. *Nat. Cell Biol.* **2011**, *13*, 1016–1023.
20. Thermo Scientific. *Thermo Scientific Open Biosystems Expression Arrest GIPZ Lentiviral shRNAmir [Technical Manual]*; Thermo Scientific: Waltham, WA, USA, 2009.
21. Chan, C.Y.; Lawrence, C.E.; Ding, Y. Structure clustering features on the Sfold Web server. *Bioinformatics* **2005**, *21*, 3926–3928.

22. Ding, Y.; Chan, C.Y.; Lawrence, C.E. Sfold web server for statistical folding and rational design of nucleic acids. *Nucleic Acids Res.* **2004**, *32*, W135–W141.
23. Nicklin, P.; Bergman, P.; Zhang, B.; Triantafellow, E.; Wang, H.; Nyfeler, B.; Yang, H.; Hild, M.; Kung, C.; Wilson, C.; et al. Bidirectional transport of amino acids regulates mTOR and autophagy. *Cell* **2009**, *136*, 521–534.
24. TransOMIC Technologies Inc. *TransEDIT™ Lentiviral gRNA plus Cas9 (pCLIP-All) Target Gene Sets [Technical Manual]*; TransOMIC Technologies Inc.: Huntsville, AL, USA, 2015.
